# Selenium and Lung Cancer: A Systematic Review and Meta Analysis

**DOI:** 10.1371/journal.pone.0026259

**Published:** 2011-11-04

**Authors:** Heidi Fritz, Deborah Kennedy, Dean Fergusson, Rochelle Fernandes, Kieran Cooley, Andrew Seely, Stephen Sagar, Raimond Wong, Dugald Seely

**Affiliations:** 1 Department of Research and Epidemiology, The Canadian College of Naturopathic Medicine, Toronto, Ontario, Canada; 2 Leslie Dan Faculty of Pharmacy, The University of Toronto, Toronto, Ontario, Canada; 3 Laboratory Medicine and Pathobiology (LMP), The University of Toronto, Toronto, Ontario, Canada; 4 Clinical Epidemiology, Ottawa Hospital Research Institute, Ottawa, Ontario, Canada; 5 Department of Surgery, Ottawa Hospital Research Institute, Ottawa, Ontario, Canada; 6 Juravinski Cancer Centre and Department of Medicine, McMaster University, Hamilton, Ontario, Canada; Karolinska Institutet, Sweden

## Abstract

**Background:**

Selenium is a natural health product widely used in the treatment and prevention of lung cancers, but large chemoprevention trials have yielded conflicting results. We conducted a systematic review of selenium for lung cancers, and assessed potential interactions with conventional therapies.

**Methods and Findings:**

Two independent reviewers searched six databases from inception to March 2009 for evidence pertaining to the safety and efficacy of selenium for lung cancers. Pubmed and EMBASE were searched to October 2009 for evidence on interactions with chemo- or radiation-therapy. In the efficacy analysis there were nine reports of five RCTs and two biomarker-based studies, 29 reports of 26 observational studies, and 41 preclinical studies. Fifteen human studies, one case report, and 36 preclinical studies were included in the interactions analysis. Based on available evidence, there appears to be a different chemopreventive effect dependent on baseline selenium status, such that selenium supplementation may reduce risk of lung cancers in populations with lower baseline selenium status (serum<106 ng/mL), but increase risk of lung cancers in those with higher selenium (≥121.6 ng/mL). Pooling data from two trials yielded no impact to odds of lung cancer, OR 0.93 (95% confidence interval 0.61–1.43); other cancers that were the primary endpoints of these trials, OR 1.51 (95%CI 0.70–3.24); and all-cause-death, OR 0.93 (95%CI 0.79–1.10). In the treatment of lung cancers, selenium may reduce cisplatin-induced nephrotoxicity and side effects associated with radiation therapy.

**Conclusions:**

Selenium may be effective for lung cancer prevention among individuals with lower selenium status, but at present should not be used as a general strategy for lung cancer prevention. Although promising, more evidence on the ability of selenium to reduce cisplatin and radiation therapy toxicity is required to ensure that therapeutic efficacy is maintained before any broad clinical recommendations can be made in this context.

## Introduction

Lung cancer is a leading cause of cancer death worldwide. The American Cancer Society projected 159,390 deaths from lung cancer in 2009, accounting for about 28% of all cancer deaths [1]. Although limiting exposure to cigarette smoke is undoubtedly the most important prevention strategy, there is a lack of evidence on chemopreventive strategies for current or former smokers. Naturally occuring antioxidants such as selenium may hold promise in this regard. Such therapies are already widely in use: a recent survey found that over 50% of lung cancer patients receiving radiation therapy used complementary and alternative medicines (CAM), with 12% reporting use of selenium specifically [2].

Selenium is an essential trace element with potent antioxidant activity mediated through its ability to increase activity of the glutathione peroxidase enzymes (GPx). Selenium has long been regarded as possessing anticancer effects based on early experiments from the early 1900s that showed regression of carcinoma and sarcoma *in vivo* [3]. Selenium been shown to inhibit DNA damage *in vitro*, and reduce pulmonary metastasis and radiation-induced carcinogenesis *in vivo* [4], [5], [6], [7]. A meta-analysis of observational studies has associated selenium levels with decreased risk of lung cancer [8]. In recent years, selenium has become controversial following results from the large Selenium and Vitamin E Cancer Prevention Trial (SELECT), which was prematurely terminated after demonstrating no effect on prostate cancer risk [9]. To date, there has been no comprehensive synthesis of evidence for the use of selenium and lung cancer.

Selenium exists in many forms. The most well studied include selenomethionine (SeMet), sodium selenite, selenium methylselenocysteine (SeMeSC), 1,4,-phenylenebis (methylene) selenocyanate (p-XSC), and methylseleninic acid (MSA). Dietary selenium is composed of selenomethionine predominantly with lesser amounts of other organic selenium compounds, although concentrations vary widely according to soil selenium content [10]. Brazil nuts are known to be a particularly concentrated source of bioavailable selenium: 2 nuts delivering an average 53 mcg selenium per day increased plasma selenium levels 64% over 12 weeks, equivalent to supplementation with 100 mcg selenium as selenomethionine, and increased GPx 8.3%, superior to selenomethionine (p = 0.032) [11]. “Selenized” or selenium-enriched yeast and selenomethionine are the forms that have been used most frequently in human trials, with selenized yeast containing 54–62% selenomethionine, <1% selenite, and small amounts of other selenocompounds [12], [13], [14].

Selenium participates in human antioxidant systems as selenocysteine (SeCys) incorporated into the various selenoproteins [15]. There are at least 25 selenoproteins known in humans, including glutathione peroxidase, thioredoxin reductase, iodothyronine deiodinase, and selenoproteins P, W, and R [16], [17]. GPx accounts for 10–30% of plasma selenium, and selenoprotein P accounts for another 50% [18]. These enzymes protect cells from free radical damage and regulate DNA transcription and cell proliferation. The glutathione and thioredoxin systems in particular have long been considered the major pathways through which selenium exerts its potential chemopreventive effect [15], while newer investigations have also suggested growth inhibitory, proapoptotic activity for selenometabolites in premalignant cells [19], Finally, selenium is also involved in thyroid function, T cell immunity, and spermatogenesis [18], and is a competitive antagonist of potentially carcinogenic heavy metals such as arsenic and cadmium [20], [21].

In order to assess the risks and benefits associated with selenium supplementation for the treatment and prevention of lung cancer, we conducted a systematic review of selenium including clinical, observational, and preclinical evidence. Also included is an assessment for potential interactions with standard chemo- and radiation- therapy.

## Methods

We searched the following electronic databases for all levels of evidence pertaining to selenium and lung cancer: Pubmed, EMBASE, CINAHL, AltHealthWatch, Cochrane, and the National Library of Science and Technology. We used a broad based MeSH and keyword approach combining clinical (lung cancer) and therapeutic (selenium) search terms: “(Selenium OR Seleno*) AND (Lung Neoplasm OR Lung Cancer OR Chemoprevention OR Chemo*).” Two searches were conducted by two independent investigators (HR and DAK): the first search was conducted in March 2009 and included all the above databases. Because the first search identified relatively few studies of interest in the databases CINAHL, AltHealth Watch, Cochrane and the National Library of Science and Technology, the second search focused solely on Pubmed and EMBASE. The second search updated the first search in August 2009, and was limited to human trials.

Screening of studies was initially conducted based on title review. In the event of uncertainty, abstracts and/or full texts were also reviewed. Only English language publications were included. Human trials had to assess the efficacy of selenium in a population of predominantly lung cancer patients for the purposes of treatment, primary or secondary prevention, reduction of side effects and toxicities associated with chemo- or radiation- therapy, or assessment of potential interactions with these therapies. Clinical surrogate studies were included if they examined endpoints directly related to lung cancer risk, pathogenesis, or objective markers assessing healthy bodily function such as hematological function in lung cancer patients. All types of lung cancers (SCLC, NSCLC, mesothelioma) were included.

To be included, observational studies had to have an objective measure of selenium status, such as serum, plasma, hair, nail, or lung tissue selenium levels, and had to examine risk of lung cancer either prospectively or be conducted in patients with lung cancer comparing selenium status to patients without cancer. Due to the high possibility for confounding, studies examining dietary intake were excluded. Preclinical studies had to be conducted in lung cancer models and had to examine either anticancer effects of selenocompounds, or their potential for interaction with conventional chemo- or radiation- therapy. Preclinical studies were categorized as “positive,” “negative,” “neutral,” or “mixed.” The term “positive” designates studies that found significant anticancer effects from at least one of the selenocompounds tested in models of lung cancer, alone or additively with other agents; “negative” designates studies that found no significant beneficial effect, nor any evidence of harm. In the absence of reported levels of significance, the authors' interpretation was used to guide classification. Studies examining surrogate markers were included only if the surrogate related directly to lung cancer risk or pathogenesis.

We piloted data extraction in duplicate to assess inter-researcher reliability. Upon completion of data extraction in duplicate for eighty percent of human level studies, there were no major inconsistencies, and further duplication of data extraction was found to be redundant. Both quality and efficacy data were extracted. Extraction sheets were prepared based on the Consolidated Standards of Reporting Trials (CONSORT) statement, the Newcastle-Ottawa scale (NOS), and the Score for Assessment of Physical Experiments on Homeopathy (SAPEH) for human trials, observational studies, and preclinical studies, respectively [22], [23], [24], [25]. RCTs were also assessed for quality using the JADAD scoring system [26].

A third search was conducted in Pubmed and EMBASE from inception to the end of October 2009 to identify articles pertaining to selenium and interactions with drugs used in lung cancer treatment and/or radiation therapy, irrespective of cancer type. Study types included clinical trials, observational studies, case reports, and preclinical studies. Data was extracted using piloted data extraction sheets and analyzed for information pertaining to pharmacokinetics and interactions.

*Statistical Analysis*. For randomized controlled trials, outcome data were pooled using random effects models weighted by the inverse variance in Comprehensive Meta-analysis Version 2, Biostat, Englewood, NJ, USA. Results are presented as odds ratios with 95% confidence intervals. Heterogeneity was assessed using the I^2^ statistic.

## Results

**Figure 1:**
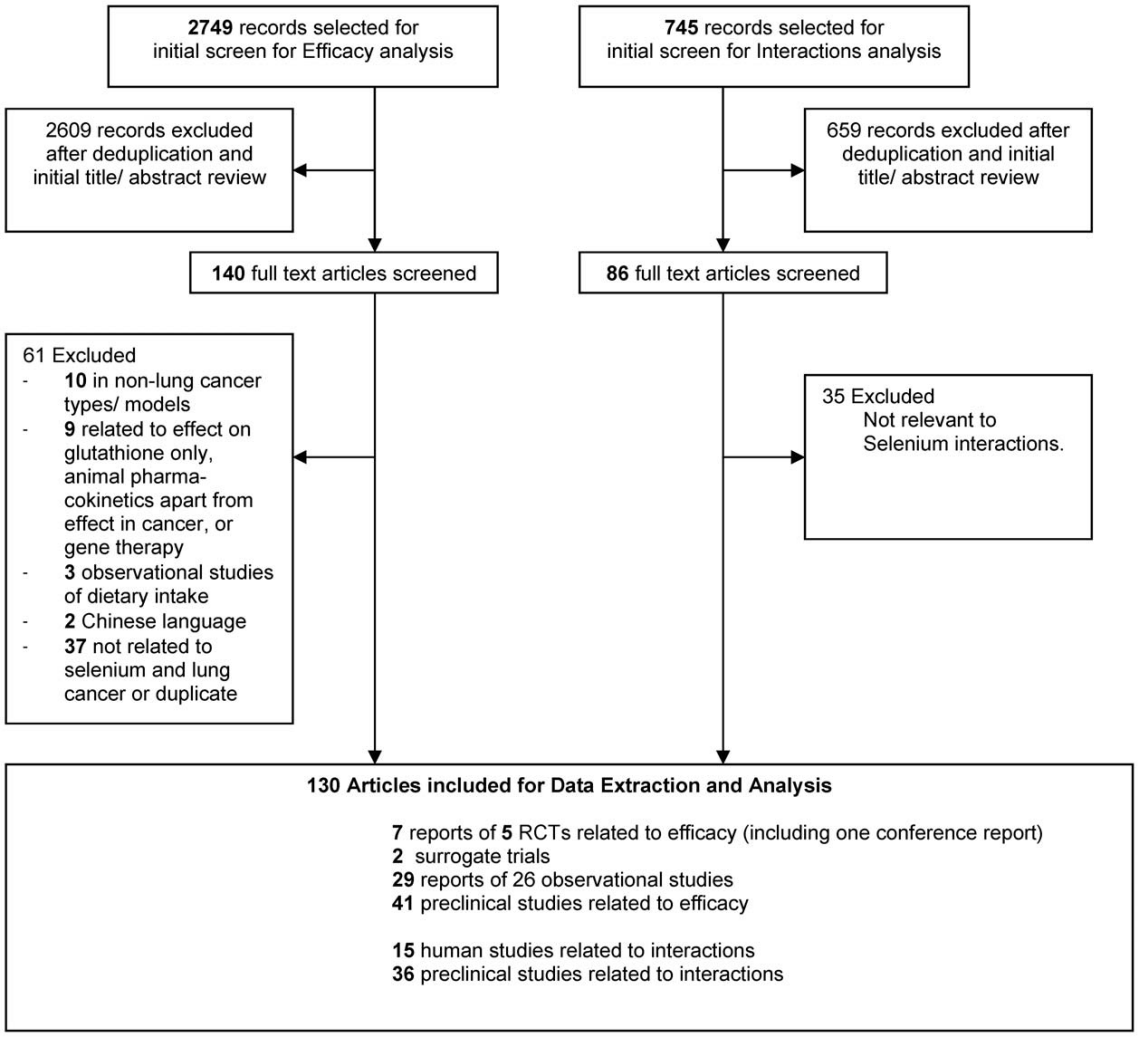
Literature Flowchart.

Of 3494 records screened, 130 records were included for full analysis and review. Seventy-eight full text articles and one conference report were selected for inclusion in the efficacy analysis. Fifty-one articles were included in the interactions analysis. **Figure 1** shows a flowchart of the literature search and study selection.

### Preclinical Evidence: Mechanism of Action and Forms of Selenium

Forty-one studies investigating the effect of selenium in preclinical models of lung cancer were included to better assess the mechanism of action through which selenium exerts its effects with respect to carcinogenesis. Of these, 37 showed results supporting an anticancer effect for selenium, two showed mixed results suggesting both pro- and anti- cancer effects, one showed negative results suggesting detriment from selenium use, and one showed no significant results in either direction. There were 12 occurrences of studies supporting a anticancer effect for selenium in vivo; ten studies found effects on cellular redox status, glutathione peroxidase, and/or thioredoxin activity; seven studies supported antiproliferative effects or growth inhibition; eight studies supported proapoptotic effects, six studies supported cytotoxic effects, four studies supported antimetastatic, anti-invasive effects; two studies showed an ability to increase survival; and one study supported an anticancer effect for selenium via potentiation of immune function. Notably, one study found mixed effects such that selenium was able to exert anticancer effects in vivo when animals were exposed to the tobacco nitrosamine NNK, but not when exposed to cigarette smoke [27]. One study found no effects on tumor growth but found that selenium decreased body weight in treated animals to 85% that of the control group [28], and one study found that selenium both increased glutathione while suppressing the cytotoxic effect of docosahexanoic acid (DHA) [29]. The three most commonly used forms of selenium were pXSC, sodium selenite, and selenomethionine/selenized yeast. **See Table S1**.

### Surrogate Trials

Trials investigating the effects of selenium supplementation on surrogate markers of lung cancer risk in at-risk populations and in lung cancer patients have shown increases in serum and tissue selenium levels, increases in glutathione, and improved immunological function. Yu found that selenium enriched rice cakes (300 mcg/d) effectively increased serum and hair selenium 178% and 194.8% respectively, increased serum glutathione peroxidase (GSH Px) 155.7%, and decreased lipid peroxide levels 74.5% compared to placebo [30]. Selenium treatment also decreased unscheduled DNA synthesis (UDS) compared to controls, indicating less DNA damage and a higher capacity for DNA repair [30]. Xu found that selenized yeast equivalent to 300 mcg/d selenium for 14 days significantly increased neutrophil oxidative metabolic activity (as measure of anti-tumor effect) and chemotaxis in lung cancer patients compared with baseline in the treatment group but not in the control group [31].

### Observational Evidence

Twenty-nine reports of 26 observational studies were included, three prospective and 23 retrospective or cross-sectional studies. Two prospective and 14 retrospective or cross-sectional studies support an inverse association between selenium status and risk of lung cancer, while one prospective and nine retrospective or cross-sectional studies showed no significant effect. These studies are listed in **Tables S2** and S**3**.

Five of the studies listed above found that higher selenium levels may also be associated with lung cancer, suggesting a possible biphasic effect for selenium or a U shaped dose-effect curve, although this evidence is somewhat weak at present. Four studies suggested a non-significant trend or non-significantly increased risk with increasing tissue selenium levels [32], [33], [34], [35], and one study found significantly increased risk of lung cancer in both the highest and the lowest quartiles of plasma selenium [16]. It is difficult to determine any clear effect beyond a certain cutoff point since the studies used different quartile cut-points, and two studies failed to report cut-points entirely. However, in the two of the studies there was non-significantly elevated risk of lung cancer in those with serum selenium between 54 to 121 ng/mL [34], and at levels >129.3 ng/mL (12.93 mcg/dL) [33]. Jablonska reported significantly increased risk of lung cancer in both those <50 ng/mL (OR 1.90, 95% CI 1.30–2.77) as well as in those with plasma selenium >90 ng/mL (OR 10.32, 95% CI 1.88–138.2), compared to those between 50–69 ng/mL [16]. See **Table S2**.

### Controlled Human Trials

Of the five RCTs included, three investigated selenium for primary prevention of lung cancer, one examined selenium for secondary prevention in resected early stage lung cancer patients, and one examined the effect of selenium in conjunction with cisplatin for the treatment of cancers including lung cancer. Pooling data from two trials, the Nutritional Prevention of Cancer (NPC) and the Selenium and Vitamin E Cancer Prevention (SELECT) trials, showed that selenium had no overall impact on odds of lung cancer, odds ratio (OR) 0.93 (95% confidence interval 0.61–1.43); odds of the other cancers that were the primary endpoints of these trials, OR 1.51 (95% CI 0.70–3.24); and odds of all cause death, OR 0.93 (95% CI 0.79–1.10). **Tables S2** and S**3** provide a description of study design and outcomes.

Of the RCTs, the Nutritional Prevention of Cancer (NPC) trial presents the strongest evidence in support of selenium supplementation to prevent cancer. The NPC study was a multicenter, randomized, double blind, placebo-controlled trial originally designed to investigate the effect of 200 mcg selenium as selenium enriched yeast for 4.5 years on non-melanoma skin cancer recurrence in 1312 subjects with a history of non-melanoma skin cancer [36]. The trial failed to show any significant effects on skin cancer, however, unexpected positive results were found for incidence of other cancers, including lung cancer. Analysis was conducted at several time points following: at 6.4 years, total cancer incidence and mortality were significantly reduced, RR 0.63 (95% CI 0.47–0.85) and RR 0.50 (0.31–0.80), respectively. Risk of lung cancer was also significantly reduced, RR 0.47 (0.22–0.98). Overall mortality was non-significantly reduced, RR 0.79 (0.61–1.02) [36], [37]. At 7.4 years, the reduction of overall cancer incidence and mortality remained significant, while there was a non-significant reduction in lung cancer risk. However, when analyzed by tertiles of baseline serum selenium, there was a 49% risk reduction in overall cancer risk in the lowest tertile (≤105.2 ng/mL), a non-significant 30% risk reduction in the middle tertile, and a non-significant 20% increased risk in the highest tertile (≥121.6 ng/mL). The benefit of selenium in the lowest tertile only remained significant at 7.9 years, HR 0.42 (0.18–0.96) [38].

Conversely, SELECT found that selenomethionine alone or in combination vitamin E had no significant effect on the risk of developing lung cancer in a setting of primary prevention: selenium, HR 1.12 (95% CI 0.73–1.72); selenium plus vitamin E, 1.16 (0.76–1.78) [9]. This study was a large, randomized, double blind, placebo controlled, multicenter study conducted in 35,533 men that investigated selenium, vitamin E, both, or placebo primarily for the prevention of prostate cancer, but included rates of lung cancer, other primary cancers, diabetes, cardiovascular events, and death as secondary outcomes. The Linxian, China chemoprevention trial also found no effect from a low dose regimen combining beta carotene, alpha-tocopherol, and selenium at doses one to two times the US recommended daily allowance (RDA) for 5.25 years [39]. In findings presented at the American Society of Clinical Oncology in 2010, Karp reported no significant effects from use of selenium on lung cancer recurrence in resected patients, with a second primary tumor rate of 1.91/4.11 per 100-person years in the selenium group and 1.36/3.66 for placebo [40]. Five year progression free survival was 72% for selenium compared to 78% for placebo [40].

Finally, a trial investigating the effect of selenium in conjunction with cisplatin chemotherapy found that selenium reduced the toxicity of chemotherapy [41]. Selenium in the form of kappa-selenocarageenan reduced leukopenia and nephrotoxicity induced by cisplatin therapy, and reduced the requirement for blood transfusions (0 vs 62+/−38 mL, p<0.05 selenium vs control group) [41].

### Safety and Therapeutic Considerations: Diabetes

Recent findings based on large trials and observational studies suggest that selenium may increase the risk of diabetes. This was reported by both the NPC and SELECT trials. The NPC trial found a 55% increased risk of diabetes among selenium supplemented subjects, HR 1.55 (95% CI 1.03–2.33) [42], while the SELECT trial reported a small non-significant increased risk of diabetes among the selenium group, RR 1.07 (99% CI 0.94–1.22) [9]. Both studies used selenium predominantly in the form of selenomethionine, whether in isolation or as selenized yeast, at doses considerably below the current safe upper limit of 400 mcg/d [43]. In the NPC trial, an exposure-response gradient was found across tertiles of baseline plasma selenium level, with risk highest in the top tertile of baseline plasma selenium level (≥121.6 ng/mL, HR 2.70, 95%CI 1.30–5.61) but non-significant findings in those below this level [42]. Karp et al failed to show an association between selenium and diabetes [40].

The NPC trial reported non-significant increased risk for melanoma, bladder cancer, breast cancer, head and neck cancer, and lymphoma and leukemia [44]. Other studies have not corroborated this association [9], [40].

### Side Effects

Signs and symptoms of selenosis (selenium toxicity) include pruritis, nail changes, brittle hair and nails, and garlic breath, and have been reported at serum selenium levels >1000 ng/mL corresponding to daily intakes >910 mcg [36], [45]. At the doses used therapeutically in intervention trials, mild side effects generally limited to dermatological and gastrointestinal symptoms have been reported [36], [46]. The SELECT trial also reported significantly increased rates of alopecia (1.28, 99%CI 1.01–1.62) and mild to moderate dermatitis (RR 1.17, 1.00–1.35) in the selenium group, but not the other groups including the one which had a combination of selenium plus vitamin E [9].

### Interactions with Chemotherapy and/or Radiotherapy

**Table 1** provides an overview of the effects of various selenium forms and chemotherapeutic drug combinations in specific cell lines *in vitro* [47], [48], [49], [50], [51], [52], [53], [54], [55]. The effects summarized include a combination index (CI) between the two substances, if reported, and when no CI was reported an account of either a positive or negative impact on neoplastic cell growth from the combination.

**Table 1:** Combination effect, *in vitro*, between forms of Selenium and Chemotherapy drugs.

	Chemotherapy	CDDP	DOC	DOX	MIC	SN38	PAX	VP-16
**Cell Line**								
**Breast**								
	MCF-7			MseA/↑ [48], [49]			ySe/± [47]	
	MDA-MB-231	S/± [50]	S/± [50]	S/± [50]	S/± [50]	S/± [50]		S/± [50]
	SK-BR-3						ySe/± [47]	
**Colon**								
	SW620					S/↑ [50]		
	Caco-2						ySe/± [47]	
	HCT116					S/↑ [50]		
**Ovarian**								
	2008						ySe/± [47]	
	Skov3R						MseA/+ [51]	
	A2780						MseA/↓ [52]	
**Prostate**								
	DU145					MseA/↑ [53]S/± [53]	MseA/↑ [53], [54]S/± [53]	MseA/↑ [53]S/± [53]
	PC3					MseA/↑ [53]S/± [53]	MseA/↑ [53], [54]ySe/± [47]S/± [53]	MseA/↑ [53]S/± [53]
	Pr14							MseA/↑ [55]S/± [55]
	Pr14C1							MseA/↑ [55]S/± [55]
	Pr111							MseA/↑ [55]S/± [55]
	LnCaP						ySe/± [47]	
**Liver**								
	HepG2						ySe/± [47]	
**Intestinal**								
	HCF8						ySe/± [47]	

**Legend**

↑ Additive effect exists where the Combination Index = 1. In the article, where the authors calculated the CI and the CI was found to be equal to 1.

+ Positive impact – where the use of the two agents in series or combination resulted in increased growth inhibition that was greater than the single agent alone.

/± No impact found.

↓ Negative impact where the use of the two agents in series or combination resulted in decreased growth inhibition versus the drug agent.

CDDP Cisplatin.

DOC Docetaxel.

DOX Doxorubicin.

MIC Mitomycin C.

MSeA Methylselenic Acid.

PAX Paclitaxel.

S Sodium selenite.

SN38 Active metabolite of irinotecan.

VP-16 Etoposide.

ySe Yeast derived selenium.

Emphasis is placed here on the pharmacokinetics changes and adverse events reported in the literature of the various selenium forms and the chemotherapy drugs used in the treatment of lung cancer.

### *In vivo* studies

**Table S4** summarizes the findings of concurrent chemotherapy and selenium supplementation *in vivo*. The most compelling evidence based on four separate studies is for protection against nephrotoxicity in cisplatin with selenium administered at least one hour prior to cisplatin [56], [57], [58], [59]. In two studies, selenium supplementation allowed for administration of higher doses of cisplatin, increased survival, and reduced cisplatin resistance [60], [61]; these findings are supported by one RCT that found reduced nephrotoxicity with concurrent use of selenium and cisplatin therapy [41].

### Clinical studies

Selenium pharmacokinetics in humans as investigated by Fakih et al are summarized in **Table 2** [62], [63]. Three studies investigated selenium supplementation concurrently with chemotherapy with varying outcomes including: reduced nephrotoxicity and leukopenia; improved response to chemotherapy, improved immune function; and lack of effect on irinotecan pharmacokinetics in one study [41], [62], [64]. **Table S5** presents a summary of these studies. None of the studies reported deleterious interactions between selenium and chemotherapy.

**Table 2:** Selenium pharmacokinetics [62], [63].

Form	Selenomethionine
Dose	2.2 mg/day
t½	183 hours (7.6 days)
S/E	Well tolerated; garlic breath
Max tolerated dose	7.2 mg/day
Blood levels necessary to protect healthy cells	15 µmol/L

### Radiotherapy

Several studies have found positive outcomes with the use of sodium selenite, dose range 200–500 mcg/day for up to 10 weeks, in reducing lymphedema [65], [66], [67], [68], [69]. Patients studied included both arm lymphedema post breast surgery and interstitial endolaryngeal edema post radiation and surgery in head and neck tumors. Radiotherapy was found to reduce selenium levels in gynecological radiation oncology, however, supplementation with sodium selenite at a dose of 500 mcg/day was found to correct this deficiency and improve antioxidant status [70], [71], [72]. In addition, supplementation with sodium selenite was found to reduce radiation induced diarrhea and improve survival [73]. A small study investigating sodium selenite at 5000 mcg/day found selenium to act as a radioprotecant for healthy cells, and reduce the incidence of mucositis and xerostomia in head and neck cancer patients receiving radiation [74].

## Discussion

The results of our review suggest that selenium supplementation may offer benefit among some individuals at risk for cancer, however its association with increased risk of diabetes warrants judicious use and further investigation of this substance. The NPC trial demonstrated that selenium supplementation may reduce risk of cancer including lung cancer among those with lower serum selenium (<106 ng/mL) [38], and a second study in lung cancer patients with low serum selenium (∼70.4 ng/mL) found beneficial effects on leukopenia, hematological toxicity, and nephrotoxicity associated with cisplatin therapy [41]. The NPC trial reported an association between with higher selenium levels (>121.6 ng/mL) and increased risk of diabetes [42], however, and this is corroborated by observational findings [75]. In the treatment of cancer, selenium may reduce toxicities and side effects associated with cisplatin and radiation therapy [41].

Several important questions around selenium supplementation and cancer prevention require further elucidation. These include determination of the dose-effect relationship, therapeutic dose, optimal selenium levels, and the best measure of selenium adequacy.

### Dose-Effect Curve

The results of the NPC trial and sixteen observational studies suggest that moderate but not very high selenium levels may reduce risk of lung cancer. In the NPC trial, subjects in the lowest tertile of serum selenium at baseline (<106 ng/ml) had significantly lower risk of lung cancer when given selenium, HR 0.42 (95%CI 0.18–0.96), while those in the highest tertile (>122 ng/ml) had non-significantly increased risk, HR 1.25 (95%CI 0.49–3.21) [38]. In addition, there were reports of non-significantly increased risk with higher selenium status or supplementation in the NPC trial and four observational studies [32], [33], [34], [35], [38], but only one observational study demonstrated this with statistical significance [16]. It therefore appears that selenium has its strongest chemopreventive effects in populations with lower baseline selenium status, and that above a certain threshold it may be of limited benefit: this was suggested by a substudy within the NPC trial: 400mcg was not more effective than placebo in reducing lung cancer risk even though it elevated serum selenium levels further than the 200mcg dose, to 250 ng/mL compared to 200 ng/mL by the 200mcg dose [12].

A 2004 meta-analysis of 16 observational also found that selenium had a protective effect primarily in populations where average selenium intake is low. Overall, relative risk of lung cancer for highest versus lowest selenium intake groups was 0.74 (95% CI 0.62–0.88) (p<0.01), with high defined as ≥100 ng/mL serum selenium or ≥55 mcg/d dietary intake [8]. However, this effect was significant only in areas where population serum levels were also low, RR 0.72 (0.56–0.93) (p<0.01), and it disappeared in areas where population selenium levels were higher (>100 mcg/L or intake >55 mcg/d) RR 0.86 (0.65–1.15) [8].

The divergent effects reported between the NPC and SELECT trials may also be explained in the context of baseline selenium status. Authors of the SELECT trial noted: “the NPC trial was conducted in men chosen for deficient levels of selenium, and found that selenium was most preventive in the men with the lowest baseline selenium levels; SELECT men generally were replete in selenium at baseline, with median serum selenium levels of 135 ng/ml vs 113 ng/ml in NPC” [9]. It should be noted that according to the Third National Health and Nutrition Examination Survey (NHANES III), average plasma selenium concentrations in the United States is 123+/−17 ng/mL [76], which places a large proportion of the US population in the high tertile according to NPC categorization.

### Effect on Diabetes

The NPC trial reported a significantly increased risk of diabetes associated with selenium supplementation which was more pronounced among those with serum selenium >121.6 ng/mL [42]. Recent evidence drawn from large cohort studies such as the Third National Health and Nutrition Examination Survey (NHANES III) has corroborated such an association between higher selenium status and diabetes, adjusted OR 7.64 (95% CI 3.34–17.46) among subjects in the highest quartile of serum selenium status (≥147 mcg/L) compared to those in the lowest quartile (<124 mcg/L) [75].

Estimates of optimal selenium intake including the Recommended Daily Allowance, currently set at 55 mcg for adults [17], are based on the dose inducing “saturation or maximization of GPx-1 activity” [43], however, several scientists have since urged reevaluation of these recommendations. It has been hypothesized that selenium may increase GPx (glutathione) activity as a “compensatory response to oxidative damage” [43] induced by selenium itself rather than as a therapeutic effect. In other words, selenium in high doses may possess pro-oxidant activity. Induction of GPx-1 may also be a mechanism through which selenium exerts its potential diabetogenic effect, since transgenic animal models overexpressing GPx-1 have been reported to experience hyperinsulinemia and insulin resistance [43]. In response to these findings, it has even been suggested that recommended selenium intakes be decreased to approximately 20mcg/d for organic selenium and less of inorganic forms [43].

Whether this recommendation can be generalized to patients receiving chemotherapy is uncertain. The uncertainty comes in light of the evidence suggesting that higher doses of selenium may benefit these patients via reductions in toxicity associated with cisplatin and radiation therapy. It is possible that higher levels of oxidative stress secondary to chemotherapy increase selenium requirements in these patients, as with other nutrients. In light of the current debate around the required amount of selenium in healthy individuals, however, it seems wise at present to limit long term, high dose selenium supplementation for the purposes of chemoprevention unless suboptimal selenium status has been established.

### Measures of Selenium Status

An important question relates to how best to establish suboptimal selenium status [43], [77]. A 2009 systematic review of methods of selenium status assessment rated all the following markers as “useful” in assessing changes in selenium status that occur over a six week or greater period in response to supplementation: plasma, RBC, and whole-blood selenium, plasma selenoprotein P, and plasma, platelet, and whole-blood GPx acitivy [18]. Serum selenium has been the most common marker used in human supplementation trials, and this is expected to be similar to plasma values. It has been argued that tissue stores such as hair and toenail selenium are more reflective of body stores over a longer period of time compared to serum levels, which are more liable to change in response to fluctuating dietary patterns, acute illness, or other stressors. RBC selenium and whole-blood selenium are reportedly more stable and may act as markers of longer term status [18].

### Forms

The biological activity of selenium in the body depends in part on its chemical form. Selenium may exist bound as a salt (eg. sodium selenite); to an amino acid (eg. selenomethionine); or as methylated forms (eg. methylselenocysteine or Se-methyl-selenocysteine) [78]. Selenomethionine can be converted to selenocysteine (SeCys) for use in selenoproteins; alternately, it can be used as a substitute for methionine in the synthesis of general proteins, potentially diverting it from its participation in anticancer pathways [79]. Inorganic salts are metabolized to selenide, which is then easily incorporated into selenoproteins such as SeCys. Finally, it has been suggested that methylated forms may be in large part responsible for selenium's anticarcinogenic effects [78], [80]. Methylated selenium such as methylselenol (CH3SeH) is formed in the selenium elimination pathway through methylation of the potentially more toxic compound dihydrogen selenide (H2Se) [79]. Alternately, methylselenol can be formed directly from the methylated selenoamino acids such as Se-methyl-selenocysteine concentrated by plant sources, such as garlic, broccoli, and certain Astragalus species, thus avoiding generation of H2Se [17], [43], [78], [79]. This may be of special relevance for individuals who have polymorphisms affecting the methylating abilities of the enzymes required for metabolizing H2Se: Ganther suggests that these individuals might “respond poorly to chemopreventive forms of Se such as inorganic salts or selenomethionine that are metabolized through the H2Se pool, but would likely show a response with Se compounds delivering Se in monomethylated forms” [79]. This has also led some to suggest that while selenomethionine and inorganic forms may be most effective for increasing selenoprotein activity, sources of methylated selenium may be most useful for reducing cancer [78].

The preclinical evidence reviewed here includes only a small amount of data on methylated selenium (methylselenic acid, methylselenol, Se-methyl-selenocysteine), however studies demonstrated proapoptotic effects [81], [82], antiproliferative effects [82], promotion of p53-mediated DNA repair [83], and protection against the carcinogen NNK [84]. The majority of preclinical studies examined inorganic selenium and the synthetic, organic selenocompound 1,4-phenylenebis(methylene) selenocyanate (pXSC). The p-XSC form showed potential in its ability to induce apoptosis [85], [86], inhibit proliferation [85], [87], and reduce tumor load *in vivo* [7], [27], [87], [88], [89], [90], [91], however it has not yet been tested in humans to our knowledge.

In human studies the most commonly used forms of selenium have been selenized yeast (NPC trial) and selenomethionine (SELECT trial). Selenized yeast contains predominantly selenomethionine (54–62%), but also small amounts of selenocysteine, γ-glutamyl-Se-methylselenocysteine, Se-methylselenocysteine, and several other forms of lower molecular weight [13], [14], [15]. Following upon the null results of the SELECT trial, it has been proposed that the more complete spectrum of monomethylated selenium species contained in selenized yeast or plant sources may be required for optimal anticancer effects [14].

### Validity of Preclinical Lung Cancer Models

Finally, we must consider the validity of preclinical models used for the investigation of anticancer effects. Chemopreventive effects of selenium have been shown in several animal models of lung cancer using NNK or B(a)P as carcinogens. These compounds are single carcinogens found in cigarette smoke, however they do not represent the full spectrum of those found in smoke, and it is likley that the collective effect of hundreds of carcinogens in cigarette smoke differs from that of single isolated carcinogens. Indeed, in the three trials using cigarette smoke as the initiating carcinogen, selenium failed to demonstrate significant protective effects [27], [28], [92]. One study directly compared the effect of selenium on NNK and cigarette smoke induced lung cancer and found efficacy in the NNK model but not the cigarette model [27].

There is conflicting evidence regarding the potential benefits of selenium for use in lung cancer chemoprevention. Evidence from the NPC trial suggests that selenium supplementation may be of benefit in the prevention of cancer in those with low selenium status (serum selenium <106 ng/ml), while supplementation may increase risk of diabetes among subjects in the higher ranges of baseline selenium status. Selenium may reduce toxicities associated with cisplatin chemotherapy and radiation therapy. Further research is required to clarify optimal dosing strategies and risks associated with use. An important limitation of this systematic review is that while comprehensive it only provides an assessment of efficacy and risk regarding the use of selenium as an individual agent rather than as part of a combined therapeutic strategy for cancer chemoprevention. Pragmatic research in the context of integrative oncology that assesses for the use of selenium and other natural health products as prescribed and taken in real world settings is required to better evaluate the additional role selenium may have for lung cancer prevention and treatment.

## Supporting Information

Table S1 ADR adriamycin; ETS environmental tobacco smoke; GPx glutathione peroxidase; KI kidney; LV liver; MSA methylseleninic acid; NNK nitrosamine 4-(methylnitrosamino)-1-(3-pyridyl)-1-butanone; NR not reported; pXSC 1,4,-phenylenebis (methylene) selenocyanate; RBC red blood cells; SeCys selenocysteine; SeMet selenomethionine; SeMeSC selenium methylselenocysteine; Se-yeast selenized yeast; MSCA 2- methyl-selenazolidine-4(R)-carboxylic acid; OSCA, 2- oxo-selenazolidine-4(R)-carboxylic acid SCA 2-unsubstituted-selenazolidine-4(R)-carboxylic acid ChSCA 2-cyclohexylselenazolidine-4-(R)-carboxylic acid Se-PBIT the selenium analog of S,S′-(1,4-phenylenebis[1,2-ethanediyl])bisisothiourea (PBIT).
(DOC)

Table S2 ATBC alpha tocopherol beta carotene trial; b/w between; CA cancer; CAD coronary artery disease; CARET carotene and retinol efficacy trial; comb combination; f/u follow up; GI gastrointestinal; LTFU loss to follow up; LuCa lung cancer; N/a not applicable; NR not reported; NSCLC non small cell lung cancer; pop population; PS performance score; pt patients; SCLC small cell lung cancer; SI smoking index (#cig/d xyr smoked); w with; yrs years.
(DOC)

Table S3 Adj adjusted; AOR adjusted odds ratio; CI confidence interval; HR hazard ratio; NMSC non melanoma skin cancer; NPC nutritional prevention of cancer trial; NR not reported; OR odds ratio; PrC prostate cancer; RBC red blood cell; RR relative risk; SELECT the selenium and vitamin E cancer prevention trial.
(DOC)

Table S4 (DOC)

Table S5 CHOP: combination chemotherapy treatment involving cyclophosphamide, doxorubicin, prednisone and vincristine. Bid: dosing frequency of two times per day. Qd: dosing frequency of once per day.
(DOC)
