# Supplementary material for: Selenium and Lung Cancer: A Systematic Review and Meta Analysis
Source: PLoS One. 2011 Nov 4;6(11):e26259. doi: 10.1371/journal.pone.0026259 (PMC3208545; doi:10.1371/journal.pone.0026259)
Supplement: Table S1 — ADR adriamycin; ETS environmental tobacco smoke; GPx glutathione peroxidase; KI kidney; LV liver; MSA methylseleninic acid; NNK nitrosamine 4-(methylnitrosamino)-1-(3-pyridyl)-1-butanone; NR not reported; pXSC 1,4,-phenylenebis (methylene) selenocyanate; RBC red blood cells; SeCys selenocysteine; SeMet selenomethionine; SeMeSC selenium methylselenocysteine; Se-yeast selenized yeast; MSCA 2- methyl-selenazolidine-4(R)-carboxylic acid; OSCA, 2- oxo-selenazolidine-4(R)-carboxylic acid SCA 2-unsubstituted-selenazolidine-4(R)-carboxylic acid ChSCA 2-cyclohexylselenazolidine-4-(R)-carboxylic acid Se-PBIT the selenium analog of S,S′-(1,4-phenylenebis[1,2-ethanediyl])bisisothiourea (PBIT). (DOC) [file pone.0026259.s001.doc]

**Table S**1. Preclinical Evidence for Selenium and Lung Cancer

| **DESIGN** | | | | | | | **OUTCOMES MEASURED & EFFECTS** | | | | | | | | |
| --- | --- | --- | --- | --- | --- | --- | --- | --- | --- | --- | --- | --- | --- | --- | --- |
| Ref.  **(Insert Endnote #)** | In vitro | In vivo | Ex vivo | N | Form | Chemo-therapy | Anticancer Effect  (↓Lung tumor incidence/ multiplicity/ volume) | ↑Survival/ ↓Mortality/ ↑time to tumor development | Antiproliferative effect/ growth inhibition | Proapoptotic effect | Anti-metastatic/ angiogenic/ invasive effect**†** | Pro-carcinogenic/ pro-angiogenic | Impact on drug effect (↑ or ↓) | **Other** | **+/**  **-/**  **n/**  **m** |
| Das 2009 [1] | y | -- | -- | n/a | Se-PBIT | n | -- | -- | y | y | -- | -- | -- | NO inhibition | + |
| Selenius 2008 [2] | y | -- | -- | n/a | sodium selenite | cisplatindocetaxel and doxorubicin | -- | -- | -- | -- | -- | -- | -- | cytotoxicity;  selenite alone was superior to selenite+drugs, but did not ↓ cytotoxicity of drugs | + |
| Poerschke 2008 [3] | y | -- | -- | n/a | SeMet, pXSC, OSCA, ChSCA, SeCys, MSA | n | -- | -- | -- | -- | -- | -- | -- | Cytotoxicity by pXSC, SeCys;  ↑ cellular thiol content by SeMet, MSA  SeCys was most pro-oxidative and cytotoxic | + |
| Shin 2007 [4] | y | -- | -- | n/a | SeMet | radiation | -- | -- | y | -- | -- | -- | -- | Selenium enhanced radiosensitivity in LuCa but not normal cells (measured as radiation induced cell death) | + |
| Romanowska 2007 [5] | y | -- | -- | n/a | sodium selenite | n | -- | -- | -- | -- | -- | -- | -- | Selenium ↑ GPx 1 and 4 (antioxidant selenoproteins) in LuCa cells | + |
| Franklin 2007 [6] | -- | y | -- | 14-15 per group | 5 selenazolidines | n | y | -- | -- | -- | -- | -- | -- | Potentially toxic: ↓ body weight for most compounds | + |
| Das 2007 [7] | -- | y | -- | 60 | synthetic organoselenocyanate: diphenylmethy selenocyanate | n | y  (lung dysplasia and hyperplasias) | -- | -- | -- | -- | -- | -- | Selenium offset loss in body weight and lipid peroxidation caused by carcinogen | + |
| Richie 2006 [8] | -- | y | -- | 150 + 184  (2 arms) | pXSC, Se-yeast | n | y  (pXSC)  n  (Se-yeast) | -- | -- | -- | -- | -- | -- | ↓ oxidative stress in lung via effects on glutathione system | + |
| El-Bayoumy 2006 [9] | y | -- | -- | n/a | pXSC | n | -- | -- | y | y | -- | -- | -- | Inhibition of COX-2 | + |
| Li 2005 [10] | -- | y | -- | 180 | selenazolidines, selenite, SeMet, SeMeSC, SeCys | n | y  (SeCys, MSCA, OSCA) | -- | -- | -- | -- | -- | -- | SeCys, SeMeSC, MSCA, SCA forms ↓the NNK-induced ↓in LV nad KI weight.  Selenium ↑GPx activity in liver, RBC, or both (d/o form). | + |
| Smith 2004 [11] | y | -- | -- | n/a | SeMet, MSA, sodium selenite | n | -- | -- | -- | -- | -- | -- | -- | SeMet and MSA promoted p53 mediated DNA repair; sodium selenite did not | + |
| Li 2004 Life Sci [12] | -- | y | -- | 145 | sodium selenite, SeMet, SeCys, SeMeSCys, MSCA, OSCA, SCA | n | -- | -- | -- | -- | -- | -- | -- | SeMet was only compound to ↑ levels of selenium in lung tissue;  ↑ GPx in RBC and liver | + |
| Li 2004 J Nutr [13] | -- | y | -- | 90 | SeMet | n | -- | -- | -- | -- | y | -- | -- | -- | + |
| Jonsson-Videsater 2004 [14] | y | -- | -- | n/a | sodium selenite | n | -- | -- | y | y | -- | -- | -- | Selenium proapoptotic in Doxorubicin resistant LuCa cells;  Induction of thioredoxin reductase | + |
| Chen 2004 [15] | y | -- | -- | n/a | selenium dioxide | n | -- | -- | y | y | -- | -- | -- | -- | + |
| Yamamoto 2003 [16] | y | -- | -- | n/a | gene therapy to ↑ amt SeMet metabolized 🡪 cytotoxic compound methylselenol | n | -- | -- | -- | y | -- | -- | -- | Therapy ↓ cell viability | + |
| Swede 2003 [17] | y | -- | -- | n/a | MSA | n | -- | -- | y | y | -- | -- | -- | Modulation of 4 cell regulatory proteins | + |
| Short 2003 [18] | y | -- | -- | n/a | selenazoladines, pXSC, SeMet, selenite, selenate | n | -- | -- | -- | -- | -- | -- | -- | Cytotoxicity (plateau’d at 75µM): selenite most toxic, then pXSC. | + |
| Das 2003 [19] | -- | y | -- | 170 | pXSC, Se-yeast | n | y  (pXSC) | -- | -- | -- | -- | -- | -- | pXSC more effective at inhibiting formation of DNA lesions | + |
| Witschi 2002 [20] | -- | y | -- | NR | pXSC | n | -- | -- | -- | -- | -- | -- | -- | No effect on cyclin D 1/2 (regulator of proliferation) | n |
| El-Bayoumy 2002 [21] | y | y | -- | 53 | pXSC | n | y | -- | y | -- | -- | -- | -- | Se ↓ Cox-2 protein levels | + |
| Bjokhem-Bergman 2002 [22] | y | -- | -- | n/a | sodium selenite; SeCys | doxorubicin | -- | -- | -- | -- | -- | -- | -- | Cytotoxicity; drug resistance cell lines were more sensitive to selenite than parent cells, but not through Pgp | + |
| Witschi 2000 Carc. [23] | -- | y | -- | NR | pXSC | n | n | -- | -- | -- | -- | -- | -- | Selenium ↓ body weight to 85% of controls | - |
| Witschi 2000  ExpLu Res [24] | -- | y | -- | 20-40 per group | pXSC | n | y (NNK)  n (ETS) | -- | -- | -- | -- | -- | -- | -- | m |
| Vadgama 2000 [25] | y | -- | -- | NR | “selenium compound” | Taxol, ADR | -- | -- | y  alone + additive w Taxol, ADR | y  additive w Taxol, ADR | -- | -- | -- | -- | + |
| Tanaka 2000 [26] | -- | y | -- | 70-77 | pXSC | n | -- | -- | -- | y | y | -- | -- | -- | + |
| Prokopczyk 2000 [27] | -- | y | -- | 140 | pXSC | n | y | -- | -- | -- | -- | -- | -- | ↑GST and GPx activity in lung | + |
| Yan 1997 [28] | -- | y | -- | 45 | sodium selenite | n | y | -- | -- | -- | y | -- | -- | no significant change in body weight | + |
| Schonberg 1997 [29] | y | -- | -- | n/a | sodium selenite and Ebselen, “a GPx mimic” | n | -- | -- | -- | -- | -- | -- | -- | Selenium (ebselen) suppressed the cytotoxic action of DHA;  Selenium ↑ GSH-Px activity in LuCa cells | m |
| Prokopczyk 1997 [30] | -- | y | -- | 200 | pXSC, SeMet | n | y | -- | -- | -- | -- | -- | -- | No effect on body weight | + |
| Kim 1997 [31] | y | -- | -- | n/a | sodium selenite | n | -- | -- | -- | -- | -- | -- | -- | Inhibition of NFκB DNA binding | + |
| Gallegos 1997 [32] | y | -- | -- | n/a | sodium selenite | n | -- | -- | -- | -- | -- | -- | -- | Cytotoxicity;  Selenium ↑ TR activity in LuCa cells | + |
| Cembryza 1997 [33] | -- | -- | y | cells from 17 patients | AE-22 Ebselen | n | -- | -- | -- | -- | -- | -- | -- | Selenium ↑ cytokine secretion from BAL cells of healthy but not of LuCa patients; selenium ↑ cytokine production from PBL cells of LuCa pts | + |
| Prokopczyk 1996 [34] | -- | y | -- | 20 | sodium selenite, pXSC | n | -- | -- | -- | -- | -- | -- | -- | Inibition of DNA adduct formation | + |
| Knizhnikov 1996 [35] | -- | y | -- | 400 | Se-yeast | following radiation | y | y | -- | -- | -- | -- | -- | Selenium administration after radiation exposure increased average lifespan and tumor latency, and ↓ incidence of malignant lung disease | + |
| El-Bayoumy 1996 [36] | -- | y | -- | 125 | pXSC | n | y | -- | -- | -- | -- | -- | -- | -- | + |
| El-Bayoumy 1993 [37] | -- | y | -- | 100 | sodium selenite, pXSC | n | y  (pXSC)  n (selenite) | -- | -- | -- | -- | -- | -- | No effect on body weight | + |
| Weiss 1992 [38] | -- | y | -- | 16-24 per group | SeMet and sodium selenite | radiation | -- | y | -- | -- | -- | -- | -- | Toxicity was lower with  SeMet | + |
| Abdullaev 1992 [39] | y | -- | -- | n/a | sodium selenite | n | -- | -- | -- | -- | -- | -- | -- | ↓ DNA and RNA synthesis (cytotoxicity), ↑ sulfhydryl compounts (eg. GSH) in tumor cells | + |
| Liu 1987 [40] | -- | y | -- | 105 | Se-yeast | n | -- | -- | -- | -- | y | -- | -- | Immunomodulation (↑ macrophage proliferation) | + |
| Blakley 1987 [41] | -- | y | -- | NR | sodium selenite | n | y | -- | -- | -- | -- | -- | -- | -- | + |
| **TOTAL** | **19** | **23** | **1** | **--** | **--** | **--** | **12** | **2** | **8** | **8** | **4** | **0** | **0** | **Cytotoxicity= 6**  **↓oxidative stress OR ↑glutathione or thioredoxin= 10** | **37 +**  **2 m**  **1 n**  **1 -** |

**References**

1. Das A, Bortner J, Desai D, Amin S, El-Bayoumy K (2009) The selenium analog of the chemopreventive compound S,S'-(1,4-phenylenebis[1,2-ethanediyl])bisisothiourea is a remarkable inducer of apoptosis and inhibitor of cell growth in human non-small cell lung cancer. Chem Biol Interact 180: 158-164.

2. Selenius M, Fernandes AP, Brodin O, Bjornstedt M, Rundlof AK (2008) Treatment of lung cancer cells with cytotoxic levels of sodium selenite: effects on the thioredoxin system. Biochem Pharmacol 75: 2092-2099.

3. Poerschke RL, Franklin MR, Moos PJ (2008) Modulation of redox status in human lung cell lines by organoselenocompounds: selenazolidines, selenomethionine, and methylseleninic acid. Toxicol In Vitro 22: 1761-1767.

4. Shin SH, Yoon MJ, Kim M, Kim JI, Lee SJ, et al. (2007) Enhanced lung cancer cell killing by the combination of selenium and ionizing radiation. Oncol Rep 17: 209-216.

5. Romanowska M, Kikawa KD, Fields JR, Maciag A, North SL, et al. (2007) Effects of selenium supplementation on expression of glutathione peroxidase isoforms in cultured human lung adenocarcinoma cell lines. Lung Cancer 55: 35-42.

6. Franklin MR, Moos PJ, El-Sayed WM, Aboul-Fadl T, Roberts JC (2007) Pre- and post-initiation chemoprevention activity of 2-alkyl/aryl selenazolidine-4(R)-carboxylic acids against tobacco-derived nitrosamine (NNK)-induced lung tumors in the A/J mouse. Chem Biol Interact 168: 211-220.

7. Das RK, Banerjee S, Bhattacharya S (2007) Amelioration of benzo (a) pyrene-induced lung carcinogenesis in strain A mice by diphenylmethyl selenocyanate. Exp Toxicol Pathol 58: 351-360.

8. Richie JP, Jr., Kleinman W, Desai DH, Das A, Amin SG, et al. (2006) The organoselenium compound 1,4-phenylenebis(methylene)selenocyanate inhibits 4-(methylnitrosamino)-1-(3-pyridyl)-1-butanone-induced tumorgenesis and enhances glutathione-related antioxidant levels in A/J mouse lung. Chem Biol Interact 161: 93-103.

9. El-Bayoumy K, Das A, Narayanan B, Narayanan N, Fiala ES, et al. (2006) Molecular targets of the chemopreventive agent 1,4-phenylenebis (methylene)-selenocyanate in human non-small cell lung cancer. Carcinogenesis 27: 1369-1376.

10. Li L, Xie Y, El-Sayed WM, Szakacs JG, Franklin MR, et al. (2005) Chemopreventive activity of selenocysteine prodrugs against tobacco-derived nitrosamine (NNK) induced lung tumors in the A/J mouse. J Biochem Mol Toxicol 19: 396-405.

11. Smith ML, Lancia JK, Mercer TI, Ip C (2004) Selenium compounds regulate p53 by common and distinctive mechanisms. Anticancer Res 24: 1401-1408.

12. Li L, Xie Y, El-Sayed WM, Szakacs JG, Roberts JC (2004) Characteristics of selenazolidine prodrugs of selenocysteine: toxicity, selenium levels, and glutathione peroxidase induction in A/J mice. Life Sci 75: 447-459.

13. Li D, Graef GL, Yee JA, Yan L (2004) Dietary supplementation with high-selenium soy protein reduces pulmonary metastasis of melanoma cells in mice. J Nutr 134: 1536-1540.

14. Jonsson-Videsater K, Bjorkhem-Bergman L, Hossain A, Soderberg A, Eriksson LC, et al. (2004) Selenite-induced apoptosis in doxorubicin-resistant cells and effects on the thioredoxin system. Biochem Pharmacol 67: 513-522.

15. Chen W-X, Cao, X.-Z., Zhu, R.-Z., Liu, W. (2004) Induction of apoptosis by SeO2 in human lung carcinoma cell line GLC-82. . Chinese Journal of Cancer Research 16(3)(pp 162-166), 2004 16: 162-166.

16. Yamamoto N, Gupta A, Xu M, Miki K, Tsujimoto Y, et al. (2003) Methioninase gene therapy with selenomethionine induces apoptosis in bcl-2-overproducing lung cancer cells. Cancer Gene Ther 10: 445-450.

17. Swede H, Dong Y, Reid M, Marshall J, Ip C (2003) Cell cycle arrest biomarkers in human lung cancer cells after treatment with selenium in culture. Cancer Epidemiol Biomarkers Prev 12: 1248-1252.

18. Short MD, Xie Y, Li L, Cassidy PB, Roberts JC (2003) Characteristics of selenazolidine prodrugs of selenocysteine: toxicity and glutathione peroxidase induction in V79 cells. J Med Chem 46: 3308-3313.

19. Das A, Desai D, Pittman B, Amin S, El-Bayoumy K (2003) Comparison of the chemopreventive efficacies of 1,4-phenylenebis(methylene)selenocyanate and selenium-enriched yeast on 4-(methylnitrosamino)-1-(3-pyridyl)-1-butanone induced lung tumorigenesis in A/J mouse. Nutr Cancer 46: 179-185.

20. Witschi H, Espiritu I, Suffia M, Pinkerton KE (2002) Expression of cyclin D1/2 in the lungs of strain A/J mice fed chemopreventive agents. Carcinogenesis 23: 289-294.

21. El-Bayoumy K, Rose DP, Papanikolaou N, Leszczynska J, Swamy MV, et al. (2002) Cyclooxygenase-2 expression influences the growth of human large and small cell lung carcinoma lines in athymic mice: impact of an organoselenium compound on growth regulation. Int J Oncol 20: 557-561.

22. Bjorkhem-Bergman L, Jonsson K, Eriksson LC, Olsson JM, Lehmann S, et al. (2002) Drug-resistant human lung cancer cells are more sensitive to selenium cytotoxicity. Effects on thioredoxin reductase and glutathione reductase. Biochem Pharmacol 63: 1875-1884.

23. Witschi H, Uyeminami D, Moran D, Espiritu I (2000) Chemoprevention of tobacco-smoke lung carcinogenesis in mice after cessation of smoke exposure. Carcinogenesis 21: 977-982.

24. Witschi H (2000) Successful and not so successful chemoprevention of tobacco smoke-induced lung tumors. Exp Lung Res 26: 743-755.

25. Vadgama JV, Wu Y, Shen D, Hsia S, Block J (2000) Effect of selenium in combination with Adriamycin or Taxol on several different cancer cells. Anticancer Research 20: 1391-1414.

26. Tanaka T, Kohno H, Murakami M, Kagami S, El-Bayoumy K (2000) Suppressing effects of dietary supplementation of the organoselenium 1,4-phenylenebis(methylene)selenocyanate and the Citrus antioxidant auraptene on lung metastasis of melanoma cells in mice. Cancer Res 60: 3713-3716.

27. Prokopczyk B, Rosa JG, Desai D, Amin S, Sohn OS, et al. (2000) Chemoprevention of lung tumorigenesis induced by a mixture of benzo(a)pyrene and 4-(methylnitrosamino)-1-(3-pyridyl)-1-butanone by the organoselenium compound 1,4-phenylenebis(methylene)selenocyanate. Cancer Lett 161: 35-46.

28. Yan L, Yee JA, McGuire MH, Graef GL (1997) Effect of dietary supplementation of selenite on pulmonary metastasis of melanoma cells in mice. Nutr Cancer 28: 165-169.

29. Schonberg SA, Rudra PK, Noding R, Skorpen F, Bjerve KS, et al. (1997) Evidence that changes in Se-glutathione peroxidase levels affect the sensitivity of human tumour cell lines to n-3 fatty acids. Carcinogenesis 18: 1897-1904.

30. Prokopczyk B, Amin S, Desai DH, Kurtzke C, Upadhyaya P, et al. (1997) Effects of 1,4-phenylenebis(methylene)selenocyanate and selenomethionine on 4-(methylnitrosamino)-1-(3-pyridyl)-1-butanone-induced tumorigenesis in A/J mouse lung. Carcinogenesis 18: 1855-1857.

31. Kim IY, Stadtman TC (1997) Inhibition of NF-kappaB DNA binding and nitric oxide induction in human T cells and lung adenocarcinoma cells by selenite treatment. Proc Natl Acad Sci U S A 94: 12904-12907.

32. Gallegos A, Berggren M, Gasdaska JR, Powis G (1997) Mechanisms of the regulation of thioredoxin reductase activity in cancer cells by the chemopreventive agent selenium. Cancer Res 57: 4965-4970.

33. Cembrzynska-Nowak M, Szklarz E, Inglot AD (1997) Modulation of cytokine production by a selenoorganic compound (AE-22) in hyperreactive or hyporeactive bronchoalveolar leukocytes of asthmatics or lung cancer patients. J Interferon Cytokine Res 17: 609-617.

34. Prokopczyk B, Cox JE, Upadhyaya P, Amin S, Desai D, et al. (1996) Effects of dietary 1,4-phenylenebis(methylene)selenocyanate on 4-(methylnitrosamino)-1-(3-pyridyl)-1-butanone-induced DNA adduct formation in lung and liver of A/J mice and F344 rats. Carcinogenesis 17: 749-753.

35. Knizhnikov VA, Shandala, N.K., Komleva, V.A., Knyazhev, V.A., Tutelyan, V.A. (1996) The effect of dietary levels of selenium on radiation resistance and radiation-induced carcinogenesis. Nutrition Research 16: 505-516.

36. el-Bayoumy K, Upadhyaya P, Desai DH, Amin S, Hoffmann D, et al. (1996) Effects of 1,4-phenylenebis(methylene)selenocyanate, phenethyl isothiocyanate, indole-3-carbinol, and d-limonene individually and in combination on the tumorigenicity of the tobacco-specific nitrosamine 4-(methylnitrosamino)-1-(3-pyridyl)-1-butanone in A/J mouse lung. Anticancer Res 16: 2709-2712.

37. el-Bayoumy K, Upadhyaya P, Desai DH, Amin S, Hecht SS (1993) Inhibition of 4-(methylnitrosamino)-1-(3-pyridyl)-1-butanone tumorigenicity in mouse lung by the synthetic organoselenium compound, 1,4-phenylenebis(methylene)selenocyanate. Carcinogenesis 14: 1111-1113.

38. Weiss JF, Srinivasan V, Kumar KS, Landauer MR (1992) Radioprotection by metals: selenium. Adv Space Res 12: 223-231.

39. Abdullaev FI, MacVicar C, Frenkel GD (1992) Inhibition by selenium of DNA and RNA synthesis in normal and malignant human cells in vitro. Cancer Lett 65: 43-49.

40. Liu Y-H, Tian, H.-S., Wang, D.-X. (1987) Inhibitory effect of selenium yeast on the metastasis of lewis lung carcinoma in C57BL mice. Studies with reference of histochemistry and ultrastructure. Chinese Medical Journal 100: 549-554.

41. Blakley BR (1987) Alterations in urethan-induced adenoma formation in mice exposed to selenium and arsenic. Drug Nutr Interact 5: 97-102.
