# Supplementary material for: Selenium and Lung Cancer: A Systematic Review and Meta Analysis
Source: PLoS One. 2011 Nov 4;6(11):e26259. doi: 10.1371/journal.pone.0026259 (PMC3208545; doi:10.1371/journal.pone.0026259)
Supplement: Table S2 — ATBC alpha tocopherol beta carotene trial; b/w between; CA cancer; CAD coronary artery disease; CARET carotene and retinol efficacy trial; comb combination; f/u follow up; GI gastrointestinal; LTFU loss to follow up; LuCa lung cancer; N/a not applicable; NR not reported; NSCLC non small cell lung cancer; pop population; PS performance score; pt patients; SCLC small cell lung cancer; SI smoking index (#cig/d xyr smoked); w with; yrs years. (DOC) [file pone.0026259.s002.doc]

**Table S**2. Methods of Controlled Human Studies of Selenium and Lung Cancer

| **Ref.** | **Population** | | | | | | | | | **Intervention** | | **Control** | **Treat-ment duration** | **Sample size** | **Study duration** | **Blinding** | **Random** | **Drop-outs / LTFU reported** | | **JADAD Score (RCTs)** |
| --- | --- | --- | --- | --- | --- | --- | --- | --- | --- | --- | --- | --- | --- | --- | --- | --- | --- | --- | --- | --- |
|  | **Population** | **Age** | **Gender** | **Smokers/ asbestos exposure** | **Staging** | **PS** | | **Previous chemo or surgery** | |
| **Primary Prevention** | |  |  |  |  |  | |  | |  | |  |  |  |  |  |  |  | |  |
| NCP Trial  Clark 1996; Duffield-Lillico 2002; Reid 2002  [1,2,3] | American | mean 63 | M 971, F 341 | n/a | previous history of basal or squamous cell carcinoma of the skin | n/a | | n/a | | Selenium as selenized yeast 200 mcg/d x mean 4.5y | | placebo | mean 4.5 y | 1312 | 7.9y | double | y | y | | **5** |
| SELECT;Lippman 2009 [4] | American, Canadian, PuertoRican | median ~62 | M 35,533  F 0 | 8-7% of each group current smokers | n/a | n/a | | n/a | | 1. Selenomethionine 200 mcg/d  2. α-tocopherol 400 IU/d  3. Selenium + vitE | | placebo | median 5.46 y | 35533 | 8 y  (stopped early; intended to be 12 y) | double | y | n (drop outs)  y (LTFU) | | **2** |
| Linxian;  Kamangar 2006 [5] | Chinese | range 40-69 | M 13,313, F 16,271 | 30% smokers | n/a | n/a | | n/a | | 2(4) factorial design: 8 tx arms in which half received ß-carotene 15mg, α-tocopherol 30mg, and selenium 50mcg in combination with other arms x 5.25 yrs | | placebo | 5.25 y | 28584 | 15 y (1986 to 2001) | double | y | NR | | **4** |
| **Secondary Prevention** | |  |  |  |  |  | |  | |  | |  |  |  |  |  |  |  | |  |
| Karp 2010 [6] | NSCLC patients | NR | NR | only 94 never smokers | stage IA and IB | NR | | complete resection surgery | | Selenium 200 mcg/d x4y  as selenized yeast | | placebo | 4 y | 1561 | median >4y f/u (stopped early) | NR | y | NR | | **2** |
| **Treatment** | |  |  |  |  |  | |  | |  | |  |  |  |  |  |  |  | |  |
| Hu 1997 [7] | Chinese patients with cancer, 27 with lung cancer | NR | M 27, F14 | NR | NR | KPS>60% | | NR | | Selenium 4000ug (Kappa-selenocarrageenan) with cisplatin chemotherapy | | cisplatin only | 8 d pre and during chemo | 41 | 2 chemotherapy cycles | NR | y | NR | | **1** |
| **Observational – Prospective** | |  |  |  | **n/a** | | **n/a** | | **n/a** | | **Measure of Selenium** | **n/a** | **Cases** | **Controls** |  | **n/a** | **n** | **n/a** | **--** | |
| Gottschall 2004 [8] | American | NR | M 79  F 0 | Asbestos exposure | -- | -- | | -- | | Plasma | |  | none | Entire cohort 79 | NR |  |  |  | |  |
| Garland 1995 [9] | Nurses from Nurse’s Health Study | range 35-55 | M 0  F  1124 | NR | -- | -- | | -- | | Toenail | |  | 505 | 619 | 41 mo:  Toenail sample in 1982, f/u 41 mo |  |  |  | |  |
| van den Brandt 1993 [10] | Dutch | range 55-69 | M 1546  F 1283 | 61% of cases and 27% controls current smokers | -- | -- | | -- | | Toenail | |  | 370 | 2457 | Data comes from period between 1986-1989: 3.3 years |  |  |  | |  |
| **Observational – Retrospective and Cross-sectional** | |  |  |  |  |  | |  | |  | |  |  |  |  |  |  |  | |  |
| Reszka 2007;  Jablonska 2008 [11,12] | Polish pts | mean ~59 | M 605  F 209 | SI: cases 750, ctl 532  (all smokers) | -- | -- | | -- | | Plasma | |  | Rezka: 404  Jablonska included: 325 | R: 410  J: 287 | 3 mo b/w pt interview and sample collection |  |  |  | |  |
| Della Rovere 2006 [13] | Italian pts | mean: cases 63, ctl 56 | M 76  F 0 | Yes, but % not reported | -- | -- | | -- | | Plasma | |  | 37 | 39 | NR |  |  |  | |  |
| Oyama 2003 [14] | Japanese NSCLC pt, pre-surgical for resection | mean 65 | M 54,  F 30 | NR | -- | -- | | -- | | Serum | |  | 84 | 0 | Surgery b/w 1991-2, with reported mean survival time 7yrs (total follow up period NR) |  |  |  | |  |
| Gromadzinska 2003 [15] | Polish LuCa pts occupationally exposed to carcinogens | mean b/w 59-60 | M 132 F 38 | SI: cases 782, ctl 543 | -- | -- | | -- | | Plasma | |  | 152 | 210 | NR |  |  |  | |  |
| Ujiie 2002 [16] | Japanese pt | NR; most pt b/w 51- 71+ yr | M 2496  F 2523 | NR | -- | -- | | -- | | Serum | |  | 2707 | 2312 | Recruited b/w Apr-Oct 1993;  Non-LuCa pt only were followed prospectively for another 5 yr |  |  |  | |  |
| Hartman 2002 [17] | Male Finnish smokers from the ATBC study | range 50-69 | M 500  F 0 | All smokers | -- | -- | | -- | | Toenail | |  | 250 | 250 | 1985-93 = 8 yrs |  |  |  | |  |
| Goodman 2001 [18] | American, CARET cohort | range 44-74 | LuCa cases and ctl: M 712 | All smokers and/ or asbestos workers | -- | -- | | -- | | Serum | |  | 365  (lung cancer arm) | 365  (lung cancer arm) | Intervention 1988-1996; f/u to 1999 =  12 yrs |  |  |  | |  |
| Ratnasinghe 2000 [19] | Chinese tin miners | mean 63 | M 324  F 0 | 70% cases, 55% ctl current smokers | -- | -- | | -- | | Serum | |  | 108 | 216 | 1992-9 =  7 yrs |  |  |  | |  |
| Knekt 1998 [20] | Finnish population | mean: ~57 | M 270  F 15 | 70% casess and 32% ctl current smokers | -- | -- | | -- | | Serum | |  | 95 | 190 | Serum samples b/w 1968-76; f/u to 1991 =  19 yrs |  |  |  | |  |
| Zachara 1997 [21] | Polish patients | mean:  cases 59  ctl 61 | M 118  F 27 | 73% cases and 36% ctl current smokers | -- | -- | | -- | | Selenium in various blood components + tumor and non tumor tissue levels | |  | 84 | 61 | 50 mo period between 1990-1995 |  |  |  | |  |
| Comstock 1997 [22] | Maryland (US) blood donors | most b/w 45-64 | M 470  F 303 | 66-74% cases, 26-32% ctl current smokers | -- | -- | | -- | | Serum | |  | 258 | 515 | 1975-1993 =  18 yrs |  |  |  | |  |
| Piccinini 1996 [23] | Untreated Italian lung and breast cancer pts (only lung described here) | mean: cases 62, ctl 57 | M 25  F 3 | NR  “matched for smoking habits” | -- | -- | | -- | | Plasma, hair | |  | 28 | 20 | NR |  |  |  | |  |
| Kabuto 1994 [24] | Japanese lung (n=77) and stomach (202) cancer pt | mean: LuCa cases and ctl 56 | M 110  F 87 (lung cancer cases + ctl only) | 67 (ctl) to 75% (cases) current or former smokers | -- | -- | | -- | | Serum | |  | 77 | 120 | Serum samples b/w 1970-72;  Diagnosis b/w 1973-83 =  13 yrs |  |  |  | |  |
| Gerhardsson 1993, 1986, 1985 [25,26,27] | Scandanavian smelter workers | mean: b/w 67-68 | M 101  F 0 | exposure to smelting metals | -- | -- | | -- | | Lung tissue | |  | 7 | 94  (smelters and non smelters) | NR: data collection began in 1975 |  |  |  | |  |
| Tominaga 1992 [28] | Japanese lung cancer pts | mean ~50 | M 46  F 16 | 23% smokers | -- | -- | | -- | | Serum | |  | 31 | 31 | July 1988 to Feb 1990 ~ 2 yrs |  |  |  | |  |
| Knekt 1990 [29] | Finnish population | range 15-99 | NR: “matched for sex” | NR | -- | -- | | -- | | Serum | |  | 1096 | 1977 | Blood samples b/w 1968-72; median f/u 10 yr =  14+ yrs |  |  |  | |  |
| Burguera 1990 [30] | Venezuelan pt | mean: cases 45, ctl 25 | M 833 (all 156 cases M),  F 679 | NR | -- | -- | | -- | | Serum | |  | 156  (22 lung cancer) | 1356 | NR |  |  |  | |  |
| Nomura 1987 [31] | Japanese residents of Hawaii | mean  62 | M 573  F 0 | NR | -- | -- | | -- | | Serum | |  | 280 (71 lung cancer) | 293 | Serum samples b/w 1971-5; f/u x 11 yrs |  |  |  | |  |
| Miyamoto 1987 [32] | Japanese pt | mean: cases ~64, family of cases ~42, ctl ~43 | M 110  F 98 | NR | -- | -- | | -- | | Serum | |  | 37 | Family of cases 115;  Unrelated ctl 56 | NR |  |  |  | |  |
| Di Ilio 1987 [33] | Italian pt w LuCa | mean: 57 | M 21  F 4 | NR | -- | -- | | -- | | Lung tumor tissue selenium,  Normal lung tissue selenium | |  | 25 | 0 | N/a : cross sectional |  |  |  | |  |
| Menkes 1986 [34] | American population | NR  ~45% b/w 55-64y | M 196  F 90 | ~ 65% current smokers in both cases and ctl | -- | -- | | -- | | Serum | |  | 99 | 196 | Blood donated 1974; f/u to 1983 =  9 yrs |  |  |  | |  |
| Salonen 1985 [35] | Eastern Finnish population | range 30-64 | M 60  F 42 | ~50% males smokers, 0% women | -- | -- | | -- | | Serum | |  | 51 | 51 | Survey 1977; f/u to 1980 =  4 yrs |  |  |  | |  |
| Chu 1984 [36] | Chinese miners | NR | M 428  F 0 | NR | -- | -- | | -- | | Whole blood | |  | 353 | 75 | NR |  |  |  | |  |

**References**

1. Clark LC, Combs GF, Jr., Turnbull BW, Slate EH, Chalker DK, et al. (1996) Effects of selenium supplementation for cancer prevention in patients with carcinoma of the skin. A randomized controlled trial. Nutritional Prevention of Cancer Study Group. Jama 276: 1957-1963.

2. Duffield-Lillico AJ, Reid ME, Turnbull BW, Combs GF, Jr., Slate EH, et al. (2002) Baseline characteristics and the effect of selenium supplementation on cancer incidence in a randomized clinical trial: a summary report of the Nutritional Prevention of Cancer Trial. Cancer Epidemiol Biomarkers Prev 11: 630-639.

3. Reid ME, Duffield-Lillico AJ, Garland L, Turnbull BW, Clark LC, et al. (2002) Selenium supplementation and lung cancer incidence: an update of the nutritional prevention of cancer trial. Cancer Epidemiol Biomarkers Prev 11: 1285-1291.

4. Lippman SM, Klein EA, Goodman PJ, Lucia MS, Thompson IM, et al. (2009) Effect of selenium and vitamin E on risk of prostate cancer and other cancers: the Selenium and Vitamin E Cancer Prevention Trial (SELECT). Jama 301: 39-51.

5. Kamangar F, Qiao YL, Yu B, Sun XD, Abnet CC, et al. (2006) Lung cancer chemoprevention: a randomized, double-blind trial in Linxian, China. Cancer Epidemiol Biomarkers Prev 15: 1562-1564.

6. D. D. Karp, S. J. Lee, G. L. Shaw Wright, D. H. Johnson, M. R. Johnston, G. E. Goodman, G. H. Clamon, G. S. Okawara, R. Marks, J. C. Ruckdeschel and MDACC Thoracic Chemoprevention Research Group. A phase III, intergroup, randomized, double-blind, chemoprevention trial of selenium (Se) supplementation in resected stage I non-small cell lung cancer (NSCLC). Journal of Clinical Oncology, 2010 ASCO Annual Meeting Proceedings, 2010. 28 (18 suppl): CRA7004.

7. Hu YJ, Chen Y, Zhang YQ, Zhou MZ, Song XM, et al. (1997) The protective role of selenium on the toxicity of cisplatin-contained chemotherapy regimen in cancer patients. Biological Trace Element Research 56: 331-341.

8. Gottschall EB, Wolfe P, Haegele AD, Zhu Z, Rose CS, et al. (2004) Increased urinary 8-isoprostaglandin F(2)alpha is associated with lower plasma selenium levels and lower vegetable and fruit intake in an asbestos-exposed cohort at risk for lung cancer. Chest 125: 83S.

9. Garland M, Morris JS, Stampfer MJ, Colditz GA, Spate VL, et al. (1995) Prospective study of toenail selenium levels and cancer among women. J Natl Cancer Inst 87: 497-505.

10. van den Brandt PA, Goldbohm RA, van 't Veer P, Bode P, Dorant E, et al. (1993) A prospective cohort study on selenium status and the risk of lung cancer. Cancer Res 53: 4860-4865.

11. Reszka E, Wasowicz W, Gromadzinska J (2007) Antioxidant defense markers modulated by glutathione S-transferase genetic polymorphism: results of lung cancer case-control study. Genes Nutr 2: 287-294.

12. Jablonska E, Gromadzinska J, Sobala W, Reszka E, Wasowicz W (2008) Lung cancer risk associated with selenium status is modified in smoking individuals by Sep15 polymorphism. Eur J Nutr 47: 47-54.

13. Della Rovere F, Granata A, Familiari D, Zirilli A, Cimino F, et al. (2006) Histamine and selenium in lung cancer. Anticancer Res 26: 2937-2942.

14. Oyama T, Kawamoto T, Matsuno K, Osaki T, Matsumoto A, et al. (2003) A case-case study comparing the usefulness of serum trace elements (Cu, Zn and Se) and tumor markers (CEA, SCC and SLX) in non-small cell lung cancer patients. Anticancer Res 23: 605-612.

15. Gromadzinska J, Wasowicz W, Rydzynski K, Szeszenia-Dabrowska N (2003) Oxidative-stress markers in blood of lung cancer patients occupationally exposed to carcinogens. Biological Trace Element Research 91: 203-215.

16. Ujiie S, Kikuchi H (2002) The relation between serum selenium value and cancer in Miyagi, Japan: 5-year follow up study. Tohoku Journal of Experimental Medicine 196: 99-109.

17. Hartman TJ, Taylor PR, Alfthan G, Fagerstrom R, Virtamo J, et al. (2002) Toenail selenium concentration and lung cancer in male smokers (Finland). Cancer Causes Control 13: 923-928.

18. Goodman GE, Schaffer S, Bankson DD, Hughes MP, Omenn GS (2001) Predictors of serum selenium in cigarette smokers and the lack of association with lung and prostate cancer risk. Cancer Epidemiol Biomarkers Prev 10: 1069-1076.

19. Ratnasinghe D, Tangrea JA, Forman MR, Hartman T, Gunter EW, et al. (2000) Serum tocopherols, selenium and lung cancer risk among tin miners in China. Cancer Causes Control 11: 129-135.

20. Knekt P, Marniemi J, Teppo L, Heliovaara M, Aromaa A (1998) Is low selenium status a risk factor for lung cancer? Am J Epidemiol 148: 975-982.

21. Zachara BA, Marchaluk-Wisniewska E, Maciag A, Peplinski J, Skokowski J, et al. (1997) Decreased selenium concentration and glutathione peroxidase activity in blood and increase of these parameters in malignant tissue of lung cancer patients. Lung 175: 321-332.

22. Comstock GW, Alberg AJ, Huang HY, Wu K, Burke AE, et al. (1997) The risk of developing lung cancer associated with antioxidants in the blood: ascorbic acid, carotenoids, alpha-tocopherol, selenium, and total peroxyl radical absorbing capacity. Cancer Epidemiol Biomarkers Prev 6: 907-916.

23. Piccinini L, Borella P, Bargellini A, Medici CI, Zoboli A (1996) A case-control study on selenium, zinc, and copper in plasma and hair of subjects affected by breast and lung cancer. Biol Trace Elem Res 51: 23-30.

24. Kabuto M, Imai H, Yonezawa C, Neriishi K, Akiba S, et al. (1994) Prediagnostic serum selenium and zinc levels and subsequent risk of lung and stomach cancer in Japan. Cancer Epidemiol Biomarkers Prev 3: 465-469.

25. Gerhardsson L, Brune D, Nordberg GF, Wester PO (1986) Selenium and other trace elements in lung tissue in smelter workers. Relationship to the occurrence of lung cancer. Acta Pharmacol Toxicol (Copenh) 59: 256-259.

26. Gerhardsson L, Brune D, Nordberg IG, Wester PO (1985) Protective effect of selenium on lung cancer in smelter workers. Br J Ind Med 42: 617-626.

27. Gerhardsson L, Nordberg GF (1993) Lung cancer in smelter workers - Interactions of metals as indicated by tissue levels. Scand J Work Environ Health 19: 90-94.

28. Tominaga K, Saito Y, Mori K, Miyazawa N, Yokoi K, et al. (1992) An evaluation of serum microelement concentrations in lung cancer and matched non-cancer patients to determine the risk of developing lung cancer: a preliminary study. Jpn J Clin Oncol 22: 96-101.

29. Knekt P, Aromaa A, Maatela J, Alfthan G, Aaran RK, et al. (1990) Serum selenium and subsequent risk of cancer among Finnish men and women. J Natl Cancer Inst 82: 864-868.

30. Burguera JL, Burguera M, Gallignani M, Alarcon OM, Burguera JA (1990) Blood serum selenium in the province of Merida, Venezuela, related to sex, cancer incidence and soil selenium content. J Trace Elem Electrolytes Health Dis 4: 73-77.

31. Nomura A, Heilbrun LK, Morris JS, Stemmermann GN (1987) Serum selenium and the risk of cancer, by specific sites: case-control analysis of prospective data. J Natl Cancer Inst 79: 103-108.

32. Miyamoto H, Araya Y, Ito M, Isobe H, Dosaka H, et al. (1987) Serum selenium and vitamin E concentrations in families of lung cancer patients. Cancer 60: 1159-1162.

33. Di Ilio C, Del Boccio G, Casaccia R, Aceto A, Di Giacomo F, et al. (1987) Selenium level and glutathione-dependent enzyme activities in normal and neoplastic human lung tissues. Carcinogenesis 8: 281-284.

34. Menkes MS, Comstock GW, Vuilleumier JP, Helsing KJ, Rider AA, et al. (1986) Serum beta-carotene, vitamins A and E, selenium, and the risk of lung cancer. N Engl J Med 315: 1250-1254.

35. Salonen JT, Salonen R, Lappetelainen R, Maenpaa PH, Alfthan G, et al. (1985) Risk of cancer in relation to serum concentrations of selenium and vitamins A and E: matched case-control analysis of prospective data. Br Med J (Clin Res Ed) 290: 417-420.

36. Chu YJ, Liu QY, Hou C, Yu SY (1984) Blood selenium concentration in residents of areas in China having a high incidence of lung cancer. Biological Trace Element Research 6: 133-137.
