# Supplementary material for: Selenium and Lung Cancer: A Systematic Review and Meta Analysis
Source: PLoS One. 2011 Nov 4;6(11):e26259. doi: 10.1371/journal.pone.0026259 (PMC3208545; doi:10.1371/journal.pone.0026259)
Supplement: Table S3 — Adj adjusted; AOR adjusted odds ratio; CI confidence interval; HR hazard ratio; NMSC non melanoma skin cancer; NPC nutritional prevention of cancer trial; NR not reported; OR odds ratio; PrC prostate cancer; RBC red blood cell; RR relative risk; SELECT the selenium and vitamin E cancer prevention trial. (DOC) [file pone.0026259.s003.doc]

**Table S**3. Outcomes of Controlled Human Studies of Selenium and Lung Cancer

| **Ref** | **Lung Cancer Incidence** | | | | | | **Other Cancer Incidence (Primary Endpoints of NPC and SELECT trials)** | | | | | **Risk Lung Cancer** | | | | **Total Cancer Incidence** | | | | | | | **Total Cancer Mortality** | | | | | **All Cause Mortality** | | | | | | | | **Lung Cancer Mortality** | | | | | |
| --- | --- | --- | --- | --- | --- | --- | --- | --- | --- | --- | --- | --- | --- | --- | --- | --- | --- | --- | --- | --- | --- | --- | --- | --- | --- | --- | --- | --- | --- | --- | --- | --- | --- | --- | --- | --- | --- | --- | --- | --- | --- |
| **RCTs**  **Primary Prevention** | **T** | | **C** | | **p value** | | **T** | **C** | | | **p value** | **Risk Measure** | | **95% CI** | **p value** | **T** | | | **C** | | | **p value** | **T** | **C** | | **p value** | | **T** | | | **C** | | | **p value** | | **Risk Measure** | | **95% CI** | | | **p value** |
| **NPC Trial**  Clark 1996 (6.4y f/u) [1] | 17/ 621 | | 31/ 629 | | 0.04 | | **NMSC:**  595/ 621  SCC 1.14 (0.93-1.39);  BCC 1.10 (0.95-1.28) | 540/ 629 | | NS (≥0.15) | | **RR** 0.54  **adj HR** 0.56 | | (0.30-0.98  (0.31-1.01) | 0.04  0.05 | 77/ 621  **RR** 0.63 (0.47-0.85)  **adj HR** 0.61 (0.46-0.82) | | | 119/ 629 | | | 0.001 for both | 29/ 621  **RR** 0.50 (0.31-0.80) | 57/ 629 | | NR, **sign** | | 108/ 621  **RR** 0.83 (0.63-1.08) | | | 129/ 629 | | | NR, sign | | **RR** 0.47 | | 0.22-0.98 | | | 0.03 |
| Duffield-Lillico 2002  (7.4y f/u of NPC trial) [2] | 25/ 621 | | 35/ 629 | | 0.18 | | -- | -- | | -- | | RR 0.70  Adj HR 0.74 | | 0.40-1.21;  0.44-1.24 | 0.18  0.26 | 105/ 621  **RR** 0.75 (0.58-0.98)  **adj HR** 0.75 (0.58-0.97) | | | 137/ 629 | | | p=0.03 for both | 40/ 621  **RR** 0.59 (0.39-0.89)  **adj HR** 0.59 (0.39-0.87) | 66/ 629 | | 0.008 for both | | -- | | | -- | | | -- | |  | |  | | |  |
| Reid 2002 (7.9y f/u of NPC trial) [3] | Analysis by tertiles of baseline selenium status | | See text | | -- | | -- | -- | | -- | | -- | | -- | -- | -- | | | -- | | | -- | -- | -- | | -- | | -- | | | -- | | | -- | | -- | | -- | | | --  -- |
| **SELECT**  Lippman 2009 [4] | Se: 75/ 8752  VitE: 67/ 8737  Both: 78/ 8703 | | 67/ 8696 | | p>0.15 | | **PrC:**  Se:432/ 8752  VitE: 473/ 8737  Both: 437/ 8703 | 416/ 8696 | | p>0.05 for all | | Se: 1.12  VitE: 1.00  Both: 1.16 | | 0.73-1.72;  0.64-1.55;  0.76-1.78 | p>0.15 | -- | | | -- | | | -- | -- | -- | | -- | | Se: 378/ 8752  VitE:  358/ 8737  Both:  359/ 8703 | | | 382/ 8696 | | | p>0.15 | | -- | | -- | | | -- |
| Kamangar 2006 [5] | -- | | -- | | -- | | -- | -- | | **--** | | Lung cancer mortality:  HR 0.98 | | 0.71-1.35 | NR | -- | | | -- | | | -- | -- | -- | | -- | | -- | | | -- | | | -- | | -- | | -- | | | --  -- |
| **Secondary Prevention** | **Fi ve Year Survival** | | | | | | **Five Year Progression Free Survival** | | | | | **Second Primary Tumor (SPT) –**  **Lung Cancer Cases**  **(number of cases/ 100 person-years)** | | | | **Second Primary Tumor (SPT) –**  **All Cancer Cases**  **(number of cases/ 100 person-years)** | | | | | | |  | | | | | | | | | | |  | | | | | | | |
|  | **T** | **C** | | | | **p value** | **T** | **C** | | **p value** | | **T** | | **C** | **p value** | **T** | | **C** | | | **p value** | |  |  |  | |  | | | | |  | |  | | |  | | |  | |
| Karp 2010 [6] | -- | -- | | | | -- | 72% | 78% | | NR | | 1.91 | | 1.36 | 0.15 | 4.11 | | 3.66 | | | 0.15 | |  |  |  | |  | | | | |  | |  | | |  | | |  | |
|  | -- | -- | | | | -- | 749/ 1041 | 406/ 520 | | -- | | -- | | -- | -- | -- | | -- | | | -- | |  |  |  | |  | | | | |  | |  | | |  | | |  | |
| **Treatment** | **Blood Transfusion Required (mL)** | | | | | | **GCSF Consumption (units)** | | | | | **WBC 10 9/ L (Bone Marrow Suppression)** | | | | **RBC count: 10 9/ L** | | | | | | | **Platelet count: 10 9/ L** | | | | **Cisplatin Induced Nephrotoxicity: Urine NAG** | | | | | | | | | | **Urine GGT** | | | | |
|  | T | | | C | | p value | T | C | | p value | | Time | T | C | p value | T | | C | | | p value | | T | C | p value | | **time** | | | **T** | | | C | | p value | | **T** | | C | | p value |
| Hu 1997 [7] | 0 | | | 62 +/-38 | | <0.05 | 110 +/- 82 | 723 +/-192 | | <0.05 | | Before Cisplatin | 651 +/-1.93 | 6.63 +/-1.73 | >0.05 | 3.60 +/- 0.65 | | 3.87 +/-0.69 | | | >0.10 | | 204.5 +/-76.7 | 210.6 +/-73.6 | >0.70 | | Before CP | | | 6.86 +/-5.8 | | | 6.35 +/-5.7 | | >0.10 | | 15.70 +/-9.47 | | 17.71 +/-9.10 | | >0.50 |
| -- |  | | |  | |  |  |  | |  | | Day 7 | 5.90 +/-1.98 | 5.29 +/-2.49 | >0.05 | 3.61 +/-0.59 | | 3.55 +/-0.62 | | | >0.50 | | 172.9 +/-73.3 | 173.3 +/-83.3 | >0.50 | | 2h | | | 9.56 +/-8.7 | | | 16.24 +/-7.33 | | <0.005 | | 18.88+/-12.04 | | 41.38+/-21.30 | | <0.05 |
| -- |  | | |  | |  |  |  | |  | | Day 10 | 4.03 +/- 1.83 | 3.56 +/- 2.08 | >0.05 | 3.32 +/- 0.59 | | 3.39 +/- 0.61 | | | >0.05 | | 121.0 +/- 49.9 | 138.8 +/- 90.9 | >0.50 | | 24h | | | 9.52 +/-6.85 | | | 13.59 +/- 6.58 | | <0.05 | | 17.18 +/-8.42 | | 17.71 +/-9.10 | | <0.001 |
| -- |  | | |  | |  |  |  | |  | | Day 14 | 3.35 +/-2.01 | 2.31 +/-1.38 | <0.05 | 3.36 +/- 0.65 | | 3.33 +/-0.59 | | | >0.80 | | 120.1 +/-55.2 | 138.7 +/-60.9 | >0.50 | | 48h | | | 8.19 +/-5.21 | | | 13.10 +/- 9.86 | | <0.01 | | 27.24 +/-12.72 | | 23.59 +/-10.51 | | <0.02 |
| -- |  | | |  | |  |  |  | |  | | Day 20 | 4.92 +/-2.16 | 4.67 +/-3.27 | >0.05 | 3.38 +/-0.61 | | 3.29 +/-0.74 | | | >0.60 | | 217.4 +/-159.4 | 179.2 +/-76.1 | >0.70 | | 72h | | | 11.18 +/-7.27 | | | 11.86 +/- 9.46 | | >0.10 | | 17.11 +/-8.37 | | 21.21 +/-10.58 | | >0.50 |
| **Prospective Cohorts** | **Risk of lung cancer** | | | | | | | | | | | **Additional Outcomes Reported** | | | | | | | | | | |  |  |  | | | |  | | | |  | |  | |  | |  | |  |
|  | Unadjusted Effect Measure | Value | | | | 95% CI | Adjusted Effect Measure | | Value | 95% CI | | Outcome | |  |  |  |  | | |  | | |  |  |  | | | |  | | | |  | |  | |  | |  | |  |
| Gottschall 2004 [8] | -- | -- | | | | -- | -- | | -- | -- | | Urinary 8 isoprostaglandin F2α (8-EPG) (marker of lipid peroxidation and positively associated with smoking) inversely related to both plasma selenium and daily fruit and vegetable consumpsion. | | | | | | | | | | |  |  |  | | | |  | | | |  | |  | |  | |  | |  |
| Garland 1995 [9] | -- | -- | | | | -- | Toenail selenium,  Adjusted RR | | 1.44 | 0.97-2.13 | | -- | |  |  |  |  | | |  | | |  |  |  | | | |  | | | |  | |  | |  | |  | |  |
| van den Brandt 1993 [10] | -- | -- | | | | -- | Toenail selenium,  Rate ratio, multivariate | | 0.50 | 0.30-0.81 | | -- | |  |  |  |  | | |  | | |  |  |  | | | |  | | | |  | |  | |  | |  | |  |
| **Retrospective and Cross-sectional** |  |  | | | |  |  | |  |  | |  | |  |  |  |  | | |  | | |  |  |  | | | |  | | | |  | |  | |  | |  | |  |
| Jablonska 2008 [11]; Reszka 2007 [12] | --  -- | --  -- | | | | --  -- | AOR, 2^nd^ quartile (Q) used as reference group:  lowest Q  highest Q  -- | | 1.90  10.32  -- | 1.30-2.77  1.88-138.2  -- | | Serum selenium, with additional analysis by genotype | |  |  |  |  | | |  | | |  |  |  | | | |  | | | |  | |  | |  | |  | |  |
| Della Rovere 2006 [13] | -- | -- | | | | -- | -- | | -- | -- | | Plasma selenium | |  |  |  |  | | |  | | |  |  |  | | | |  | | | |  | |  | |  | |  | |  |
| Gromadzinska 2003 [14] | OR, low quartile compared to high | 3.047 | | | | NR  p<0.0001 | -- | | -- | -- | | -- | |  |  |  |  | | |  | | |  |  |  | | | |  | | | |  | |  | |  | |  | |  |
| Oyama 2003 [15] | -- | -- | | | | -- | -- | | -- | -- | | Serum selenium | |  |  |  |  | | |  | | |  |  |  | | | |  | | | |  | |  | |  | |  | |  |
| Ujiie 2002 [16] | OR, total cancer, lowest to highest tertile | 2.52 | | | | 2.18-2.90 | -- | | -- | -- | | Serum selenium | |  |  |  |  | | |  | | |  |  |  | | | |  | | | |  | |  | |  | |  | |  |
| Hartman 2002 [17] | -- | -- | | | | -- | AOR, for those randomized “earliest” vs  later during the trial | | 0.20  0.61 | 0.09-0.44  0.27-1.41 | | toenail selenium | |  |  |  |  | | |  | | |  |  |  | | | |  | | | |  | |  | |  | |  | |  |
| Goodman 2001 [18] | -- | -- | | | | -- | AOR | | 1.20 | 0.77-1.88 | | -- | |  |  |  |  | | |  | | |  |  |  | | | |  | | | |  | |  | |  | |  | |  |
| Ratnasinghe 2000 [19] | -- | -- | | | | -- | AOR | | 1.2 | 0.6-2.4 | | -- | |  |  |  |  | | |  | | |  |  |  | | | |  | | | |  | |  | |  | |  | |  |
| Knekt 1998 [20] | -- | -- | | | | -- | AOR by gender | | M 0.30  F 0.0 | p trend  0.001  0.897 | | Serum selenium | |  |  |  |  | | |  | | |  |  |  | | | |  | | | |  | |  | |  | |  | |  |
| Zachara 1997 [21] | -- | -- | | | | -- | -- | | -- | -- | | Plasma, whole blood, and RBC selenium levels | |  |  |  |  | | |  | | |  |  |  | | | |  | | | |  | |  | |  | |  | |  |
| Comstock 1997 [22] | OR among controls | 0.65 | | | | NR | -- | | -- | -- | | -- | |  |  |  |  | | |  | | |  |  |  | | | |  | | | |  | |  | |  | |  | |  |
| Piccinini 1996 [23] | -- | -- | | | | -- | -- | | -- | -- | | Hair selenium | |  |  |  |  | | |  | | |  |  |  | | | |  | | | |  | |  | |  | |  | |  |
| Kabuto 1994 [24] | -- | -- | | | | -- | AOR (lowest quartile compared to highest) | | 1.8 | 0.7-5.0 | | -- | |  |  |  |  | | |  | | |  |  |  | | | |  | | | |  | |  | |  | |  | |  |
| Gerhardsson 1993, 1986, 1985 [25,26,27] | -- | -- | | | | -- | -- | | -- | -- | | Lung tissue selenium levels | |  |  |  |  | | |  | | |  |  |  | | | |  | | | |  | |  | |  | |  | |  |
| Tominaga 1992 [28] | RR (lowest compared to highest tertile) | 2.84 | | | | 0.79-10.18 | -- | | -- | -- | | -- | |  |  |  |  | | |  | | |  |  |  | | | |  | | | |  | |  | |  | |  | |  |
| Knekt 1990 [29] | -- | -- | | | | -- | Adjusted RR | | 0.41 | 0.17-0.94 | | -- | |  |  |  |  | | |  | | |  |  |  | | | |  | | | |  | |  | |  | |  | |  |
| Burguera 1990 [30] | -- | -- | | | | -- | -- | | -- | -- | | Serum selenium | |  |  |  |  | | |  | | |  |  |  | | | |  | | | |  | |  | |  | |  | |  |
| Nomura 1987 [31] | -- | -- | | | | -- | Adjusted RR, lowest to highest quintile | | 1.1 | p trend 0.46 | | Serum selenium | |  |  |  |  | | |  | | |  |  |  | | | |  | | | |  | |  | |  | |  | |  |
| Miyamoto 1987 [32] | -- | -- | | | | -- | -- | | -- | -- | | Serum selenium | |  |  |  |  | | |  | | |  |  |  | | | |  | | | |  | |  | |  | |  | |  |
| Di Ilio 1987 [33] | -- | -- | | | | -- | -- | | -- | -- | | Lung tumor selenium level and glutathione activity | |  |  |  |  | | |  | | |  |  |  | | | |  | | | |  | |  | |  | |  | |  |
| Menkes 1986 [34] | OR (lowest quintile compared to highest) | 3.33 | | | | NR | -- | | -- | -- | | -- | |  |  |  |  | | |  | | |  |  |  | | | |  | | | |  | |  | |  | |  | |  |
| Salonen 1985 [35] | -- | -- | | | | -- | Adjusted RR for serum selenium ≤47 mcg/l (all cancer mortality) | | 5.8 | 1.2-29.0 | | -- | |  |  |  |  | | |  | | |  |  |  | | | |  | | | |  | |  | |  | |  | |  |
| Chu 1984 [36] | -- | -- | | | | -- | -- | | -- | -- | | Serum selenium | |  |  |  |  | | |  | | |  |  |  | | | |  | | | |  | |  | |  | |  | |  |

**References**

1. Clark LC, Combs GF, Jr., Turnbull BW, Slate EH, Chalker DK, et al. (1996) Effects of selenium supplementation for cancer prevention in patients with carcinoma of the skin. A randomized controlled trial. Nutritional Prevention of Cancer Study Group. Jama 276: 1957-1963.

2. Duffield-Lillico AJ, Reid ME, Turnbull BW, Combs GF, Jr., Slate EH, et al. (2002) Baseline characteristics and the effect of selenium supplementation on cancer incidence in a randomized clinical trial: a summary report of the Nutritional Prevention of Cancer Trial. Cancer Epidemiol Biomarkers Prev 11: 630-639.

3. Reid ME, Duffield-Lillico AJ, Garland L, Turnbull BW, Clark LC, et al. (2002) Selenium supplementation and lung cancer incidence: an update of the nutritional prevention of cancer trial. Cancer Epidemiol Biomarkers Prev 11: 1285-1291.

4. Lippman SM, Klein EA, Goodman PJ, Lucia MS, Thompson IM, et al. (2009) Effect of selenium and vitamin E on risk of prostate cancer and other cancers: the Selenium and Vitamin E Cancer Prevention Trial (SELECT). Jama 301: 39-51.

5. Kamangar F, Qiao YL, Yu B, Sun XD, Abnet CC, et al. (2006) Lung cancer chemoprevention: a randomized, double-blind trial in Linxian, China. Cancer Epidemiol Biomarkers Prev 15: 1562-1564.

6. D. D. Karp, S. J. Lee, G. L. Shaw Wright, D. H. Johnson, M. R. Johnston, G. E. Goodman, G. H. Clamon, G. S. Okawara, R. Marks, J. C. Ruckdeschel and MDACC Thoracic Chemoprevention Research Group. A phase III, intergroup, randomized, double-blind, chemoprevention trial of selenium (Se) supplementation in resected stage I non-small cell lung cancer (NSCLC). Journal of Clinical Oncology, 2010 ASCO Annual Meeting Proceedings, 2010. 28 (18 suppl): CRA7004.

7. Hu YJ, Chen Y, Zhang YQ, Zhou MZ, Song XM, et al. (1997) The protective role of selenium on the toxicity of cisplatin-contained chemotherapy regimen in cancer patients. Biological Trace Element Research 56: 331-341.

8. Gottschall EB, Wolfe P, Haegele AD, Zhu Z, Rose CS, et al. (2004) Increased urinary 8-isoprostaglandin F(2)alpha is associated with lower plasma selenium levels and lower vegetable and fruit intake in an asbestos-exposed cohort at risk for lung cancer. Chest 125: 83S.

9. Garland M, Morris JS, Stampfer MJ, Colditz GA, Spate VL, et al. (1995) Prospective study of toenail selenium levels and cancer among women. J Natl Cancer Inst 87: 497-505.

10. van den Brandt PA, Goldbohm RA, van 't Veer P, Bode P, Dorant E, et al. (1993) A prospective cohort study on selenium status and the risk of lung cancer. Cancer Res 53: 4860-4865.

11. Jablonska E, Gromadzinska J, Sobala W, Reszka E, Wasowicz W (2008) Lung cancer risk associated with selenium status is modified in smoking individuals by Sep15 polymorphism. Eur J Nutr 47: 47-54.

12. Reszka E, Wasowicz W, Gromadzinska J (2007) Antioxidant defense markers modulated by glutathione S-transferase genetic polymorphism: results of lung cancer case-control study. Genes Nutr 2: 287-294.

13. Della Rovere F, Granata A, Familiari D, Zirilli A, Cimino F, et al. (2006) Histamine and selenium in lung cancer. Anticancer Res 26: 2937-2942.

14. Gromadzinska J, Wasowicz W, Rydzynski K, Szeszenia-Dabrowska N (2003) Oxidative-stress markers in blood of lung cancer patients occupationally exposed to carcinogens. Biological Trace Element Research 91: 203-215.

15. Oyama T, Kawamoto T, Matsuno K, Osaki T, Matsumoto A, et al. (2003) A case-case study comparing the usefulness of serum trace elements (Cu, Zn and Se) and tumor markers (CEA, SCC and SLX) in non-small cell lung cancer patients. Anticancer Res 23: 605-612.

16. Ujiie S, Kikuchi H (2002) The relation between serum selenium value and cancer in Miyagi, Japan: 5-year follow up study. Tohoku Journal of Experimental Medicine 196: 99-109.

17. Hartman TJ, Taylor PR, Alfthan G, Fagerstrom R, Virtamo J, et al. (2002) Toenail selenium concentration and lung cancer in male smokers (Finland). Cancer Causes Control 13: 923-928.

18. Goodman GE, Schaffer S, Bankson DD, Hughes MP, Omenn GS (2001) Predictors of serum selenium in cigarette smokers and the lack of association with lung and prostate cancer risk. Cancer Epidemiol Biomarkers Prev 10: 1069-1076.

19. Ratnasinghe D, Tangrea JA, Forman MR, Hartman T, Gunter EW, et al. (2000) Serum tocopherols, selenium and lung cancer risk among tin miners in China. Cancer Causes Control 11: 129-135.

20. Knekt P, Marniemi J, Teppo L, Heliovaara M, Aromaa A (1998) Is low selenium status a risk factor for lung cancer? Am J Epidemiol 148: 975-982.

21. Zachara BA, Marchaluk-Wisniewska E, Maciag A, Peplinski J, Skokowski J, et al. (1997) Decreased selenium concentration and glutathione peroxidase activity in blood and increase of these parameters in malignant tissue of lung cancer patients. Lung 175: 321-332.

22. Comstock GW, Alberg AJ, Huang HY, Wu K, Burke AE, et al. (1997) The risk of developing lung cancer associated with antioxidants in the blood: ascorbic acid, carotenoids, alpha-tocopherol, selenium, and total peroxyl radical absorbing capacity. Cancer Epidemiol Biomarkers Prev 6: 907-916.

23. Piccinini L, Borella P, Bargellini A, Medici CI, Zoboli A (1996) A case-control study on selenium, zinc, and copper in plasma and hair of subjects affected by breast and lung cancer. Biol Trace Elem Res 51: 23-30.

24. Kabuto M, Imai H, Yonezawa C, Neriishi K, Akiba S, et al. (1994) Prediagnostic serum selenium and zinc levels and subsequent risk of lung and stomach cancer in Japan. Cancer Epidemiol Biomarkers Prev 3: 465-469.

25. Gerhardsson L, Brune D, Nordberg GF, Wester PO (1986) Selenium and other trace elements in lung tissue in smelter workers. Relationship to the occurrence of lung cancer. Acta Pharmacol Toxicol (Copenh) 59: 256-259.

26. Gerhardsson L, Brune D, Nordberg IG, Wester PO (1985) Protective effect of selenium on lung cancer in smelter workers. Br J Ind Med 42: 617-626.

27. Gerhardsson L, Nordberg GF (1993) Lung cancer in smelter workers - Interactions of metals as indicated by tissue levels. Scand J Work Environ Health 19: 90-94.

28. Tominaga K, Saito Y, Mori K, Miyazawa N, Yokoi K, et al. (1992) An evaluation of serum microelement concentrations in lung cancer and matched non-cancer patients to determine the risk of developing lung cancer: a preliminary study. Jpn J Clin Oncol 22: 96-101.

29. Knekt P, Aromaa A, Maatela J, Alfthan G, Aaran RK, et al. (1990) Serum selenium and subsequent risk of cancer among Finnish men and women. J Natl Cancer Inst 82: 864-868.

30. Burguera JL, Burguera M, Gallignani M, Alarcon OM, Burguera JA (1990) Blood serum selenium in the province of Merida, Venezuela, related to sex, cancer incidence and soil selenium content. J Trace Elem Electrolytes Health Dis 4: 73-77.

31. Nomura A, Heilbrun LK, Morris JS, Stemmermann GN (1987) Serum selenium and the risk of cancer, by specific sites: case-control analysis of prospective data. J Natl Cancer Inst 79: 103-108.

32. Miyamoto H, Araya Y, Ito M, Isobe H, Dosaka H, et al. (1987) Serum selenium and vitamin E concentrations in families of lung cancer patients. Cancer 60: 1159-1162.

33. Di Ilio C, Del Boccio G, Casaccia R, Aceto A, Di Giacomo F, et al. (1987) Selenium level and glutathione-dependent enzyme activities in normal and neoplastic human lung tissues. Carcinogenesis 8: 281-284.

34. Menkes MS, Comstock GW, Vuilleumier JP, Helsing KJ, Rider AA, et al. (1986) Serum beta-carotene, vitamins A and E, selenium, and the risk of lung cancer. N Engl J Med 315: 1250-1254.

35. Salonen JT, Salonen R, Lappetelainen R, Maenpaa PH, Alfthan G, et al. (1985) Risk of cancer in relation to serum concentrations of selenium and vitamins A and E: matched case-control analysis of prospective data. Br Med J (Clin Res Ed) 290: 417-420.

36. Chu YJ, Liu QY, Hou C, Yu SY (1984) Blood selenium concentration in residents of areas in China having a high incidence of lung cancer. Biological Trace Element Research 6: 133-137.
