# Supplementary material for: Selenium and Lung Cancer: A Systematic Review and Meta Analysis
Source: PLoS One. 2011 Nov 4;6(11):e26259. doi: 10.1371/journal.pone.0026259 (PMC3208545; doi:10.1371/journal.pone.0026259)
Supplement: Table S4 — (DOC) [file pone.0026259.s004.doc]

**Table S4: *In vivo* Findings for Selenium and Interactions with Chemotherapy Drugs**

| **Chemotherapy** | **Cancer** | **Form** | **Impact** | **Reference** |
| --- | --- | --- | --- | --- |
| Cyclophosphamide | Ehrlich ascites | Diphenylmethyl selenocyanate | - Synergistically enhanced the efficacy - Reduced the impact of cyclophosphamide on hemopoietic system | Chakraborty 2009 [1] |
|  | n/a | Ebselen | - Reduced genotoxic effect on healthy cells (DNA, lipids and proteins) | Tripathi 2008 [2] |
| Cisplatin | Yolk sac | Sodium selenite | - Reduction of nephrotoxicity, myeloid suppression and body weight loss - 1.9 fold increase in LD50 dose | Ohkawal 1988 [3] |
|  | Breast cancer cells  Plasmacytoma | Sodium selenite | - Reduced nephrotoxicity when given in advance of cisplatin | Baldew 1989 [4]  Vermeulen 1993 [5] |
|  | Melanoma | Selenomethionine | - Reduced nephrotoxicity when given in advance of cisplatin | Rau 1998 [6] |
|  | Colon | Sodium selenite | - Increased the dose of cisplatin administered, providing increased survival | Satoh 1992 [7] |
|  | Ovarian | Selenite  Selenomethionine | - Both forms have the potential to reduce cisplatin induced drug resistance | Caffery 2000 [8] |
| Doxorubicin | Head and neck tumor | Methylselenocysteine (MSC) | - Demonstrated antiangiogenic effects and enhanced drug delivery | Bhattacharya 2008 [9] |
|  | Breast | Methyl selenic acid (MSeA) | - Synergistic effect | Li 2007 [10] |
| Irinotecan | Head and neck tumor | Methylselenocysteine (MSC) | - Demonstrated antiangiogenic effects | Yin 2006 [11] |
|  | Head and neck tumor | Methylselenocysteine (MSC) | - Increase intratumor area under the curve of SN-38 (metabolite of irinotecan) and decreased efflux pump of SN-38 | Azrak 2007 [12] |
|  | Head and neck tumor | Methylselenocysteine (MSC) | - Protection and enhanced anti tumor effects are MSC dose dependent with irinotecan | Azrak 2007 [12] |
|  | A253  Head and neck tumor  HCT-8  HT-29 | Methylselenocysteine (MSC) | - Higher cured rates achieved and reduced toxicity with MSC | Cao 2004 [13] |
| SN-38 (metabolite of irinotecan) | Prostate | Methyl selenic acid  Sodium selenite | - JNK activation is crucial in apoptosis induced by SN-38/MSeA - Sodium selenite found not to be effective | Hu 2005 [14] |
| Etoposide | Prostate | Methyl selenic acid (MSeA)  Sodium selenite | - Potentiates the effect of etoposide | Gonzalez 2007 [15] |

**References**

1. Chakraborty P, Sk UH, Bhattacharya S (2009) Chemoprotection and enhancement of cancer chemotherapeutic efficacy of cyclophosphamide in mice bearing Ehrlich ascites carcinoma by diphenylmethyl selenocyanate. Cancer Chemotherapy & Pharmacology 64: 971-980.

2. Tripathi DN, Jena GB (2008) Ebselen attenuates cyclophosphamide-induced oxidative stress and DNA damage in mice. Free Radical Research 42: 966-977.

3. Ohkawa K, Tsukada Y, Dohzono H, Koike K, Terashima Y (1988) The effects of co-administration of selenium and cis-platin (CDDP) on CDDP-induced toxicity and antitumour activity. British Journal of Cancer 58: 38-41.

4. Baldew GS, van den Hamer CJ, Los G, Vermeulen NP, de Goeij JJ, et al. (1989) Selenium-induced protection against cis-diamminedichloroplatinum(II) nephrotoxicity in mice and rats. Cancer Research 49: 3020-3023.

5. Vermeulen NP, Baldew GS, Los G, McVie JG, De Goeij JJ (1993) Reduction of cisplatin nephrotoxicity by sodium selenite. Lack of interaction at the pharmacokinetic level of both compounds. Drug Metabolism & Disposition 21: 30-36.

6. Rao M, Rao MN (1998) Protective effects of selenomethionine against cisplatin-induced renal toxicity in mice and rats. Journal of Pharmacy & Pharmacology 50: 687-691.

7. Satoh M, Naganuma A, Imura N (1992) Effect of coadministration of selenite on the toxicity and antitumor activity of cis-diamminedichloroplatinum (II) given repeatedly to mice. Cancer Chemotherapy & Pharmacology 30: 439-443.

8. Caffrey PB, Frenkel GD (2000) Selenium compounds prevent the induction of drug resistance by cisplatin in human ovarian tumor xenografts in vivo. Cancer Chemotherapy & Pharmacology 46: 74-78.

9. Bhattacharya A, Seshadri M, Oven SD, Toth K, Vaughan MM, et al. (2008) Tumor vascular maturation and improved drug delivery induced by methylselenocysteine leads to therapeutic synergy with anticancer drugs. Clinical Cancer Research 14: 3926-3932.

10. Li S, Zhou Y, Wang R, Zhang H, Dong Y, et al. (2007) Selenium sensitizes MCF-7 breast cancer cells to doxorubicin-induced apoptosis through modulation of phospho-Akt and its downstream substrates. Molecular Cancer Therapeutics 6: 1031-1038.

11. Yin MB, Li ZR, Toth K, Cao S, Durrani FA, et al. (2006) Potentiation of irinotecan sensitivity by Se-methylselenocysteine in an in vivo tumor model is associated with downregulation of cyclooxygenase-2, inducible nitric oxide synthase, and hypoxia-inducible factor 1alpha expression, resulting in reduced angiogenesis. Oncogene 25: 2509-2519.

12. Azrak RG, Cao S, Pendyala L, Durrani FA, Fakih M, et al. (2007) Efficacy of increasing the therapeutic index of irinotecan, plasma and tissue selenium concentrations is methylselenocysteine dose dependent. Biochemical Pharmacology 73: 1280-1287.

13. Cao S, Durrani FA, Rustum YM (2004) Selective modulation of the therapeutic efficacy of anticancer drugs by selenium containing compounds against human tumor xenografts. Clinical Cancer Research 10: 2561-2569.

14. Hu H, Jiang C, Ip C, Rustum YM, Lu J (2005) Methylseleninic acid potentiates apoptosis induced by chemotherapeutic drugs in androgen-independent prostate cancer cells. Clinical Cancer Research 11: 2379-2388.

15. Gonzalez-Moreno O, Segura V, Serrano D, Nguewa P, De Las Rivas J, et al. (2007) Methylseleninic acid enhances the effect of etoposide to inhibit prostate cancer growth in vivo. International Journal of Cancer 121: 1197-1204.
