# Supplementary material for: Selenium and Lung Cancer: A Systematic Review and Meta Analysis
Source: PLoS One. 2011 Nov 4;6(11):e26259. doi: 10.1371/journal.pone.0026259 (PMC3208545; doi:10.1371/journal.pone.0026259)
Supplement: Table S5 — CHOP: combination chemotherapy treatment involving cyclophosphamide, doxorubicin, prednisone and vincristine. Bid: dosing frequency of two times per day. Qd: dosing frequency of once per day. (DOC) [file pone.0026259.s005.doc]

**Table S5: Human Studies of Selenium Supplementation Concurrent with Chemotherapy Results**

| **Author** | **Type of cancer** | **Chemotherapy** | **Dose** | **Form** | **Outcome** |
| --- | --- | --- | --- | --- | --- |
| Hu 1997 [1] | Solid tumour, various | Cisplatin 60-80 mg/m^2^ | 4 mg/day for 8 days | Seleno-kappacarrageenan | - Reduced nephrotoxicity and leucopenia |
| Asfour 2007 [2] | Non Hodgkin’s Lymphoma | Standard CHOP | 0.2 mg/kg/day for 30 days | Sodium selenite | - Improved response to chemotherapy, - Immune system support |
| Fakih 2008 [3] | Solid tumors, various | Irinotecan  125 mg/m^2^ | 3.2 mg to 7.2 mg bid for 7 days;  3.2 mg to 7.2 mg qd for 28 days; | Selenomethionine | - No impact on irinotecan pharmacokinetics |

**References**

1. Hu YJ, Chen Y, Zhang YQ, Zhou MZ, Song XM, et al. (1997) The protective role of selenium on the toxicity of cisplatin-contained chemotherapy regimen in cancer patients. Biological Trace Element Research 56: 331-341.

2. Asfour IA, Fayek M, Raouf S, Soliman M, Hegab HM, et al. (2007) The impact of high-dose sodium selenite therapy on Bcl-2 expression in adult non-Nodgkin's lymphoma patients: Correlation with response and survival. Biological Trace Element Research 120: 1-10.

3. Fakih MG, Pendyala L, Brady W, Smith PF, Ross ME, et al. (2008) A Phase I and pharmacokinetic study of selenomethionine in combination with a fixed dose of irinotecan in solid tumors. Cancer Chemotherapy & Pharmacology 62: 499-508.
